# Robust Stoichiometry of FliW-CsrA Governs Flagellin Homeostasis and Cytoplasmic Organization in Bacillus subtilis

**DOI:** 10.1128/mBio.00533-19

**Published:** 2019-05-21

**Authors:** R. T. Oshiro, S. Rajendren, H. A. Hundley, D. B. Kearns

**Affiliations:** a Department of Biology, Indiana University Bloomington, Bloomington, Indiana, USA; b Department of Medical Sciences, Indiana University Bloomington, Bloomington, Indiana, USA; Princeton University

**Keywords:** FtsZ, flagella, *fliC*, homeostasis, minicell, motility, toxin-antitoxin

## Abstract

Flagellin (Hag) is one of the most abundant proteins in Bacillus subtilis. Here we show that each flagellar filament is assembled from ∼12,000 Hag monomers and that there is a cytoplasmic pool of Hag that is restricted to 5% of the total. Hag is thought to be restricted at the level of translation by a partner-switching mechanism involving FliW and the homodimeric RNA-binding protein CsrA (CsrA^dimer^). We further show that the mechanism of translation inhibition is hypersensitive due to a 1:1 ratio of Hag to FliW, a 1:1 inhibitory ratio of FliW to CsrA^dimer^, and a nearly 1:1 ratio of CsrA^dimer^ to *hag* transcripts. Equimolarity of all components couples single-molecule detection of Hag export to compensatory translation and causes cytoplasmic Hag concentrations to oscillate around the level of FliW. We found that stoichiometry is ensured by genetic architecture, translational coupling, and the ability of CsrA^dimer^ to restrict *hag* transcript accumulation. We further show that homeostasis prevents Hag hyperaccumulation that would otherwise cause severe defects in intracellular architecture, perhaps due to increased molecular crowding. We note that FliW-CsrA-mediated structural homeostasis has similarities to that seen with some toxin-antitoxin systems.

## Abstract (precis)

The intracellular concentration of flagellar filament protein Hag is restricted by the Hag-FliW-CsrA system in B. subtilis. Here we show that the Hag-FliW-CsrA^dimer^ system functions at nearly 1:1:1 stoichiometry and that the system is both robust with respect to perturbation and hypersensitive to the Hag intracellular concentration. Moreover, restriction of cytoplasmic Hag levels is important for maintaining proper intracellular architecture, as artificial Hag hyperaccumulation led to generalized spatial defects and a high frequency of minicell production. The Hag-FliW-CsrA system is conserved in the deeper branches of bacterial phylogeny, and we note that the Hag-FliW-CsrA “homeostasis module” resembles a toxin-antitoxin system where, by analogy, CsrA is the “toxin,” FliW is the “antitoxin,” and Hag is the target.

## INTRODUCTION

Homeostasis is the process in which a dynamic system is held in robust equilibrium to avoid extreme states that could be potentially wasteful, inefficient, or toxic. Negative feedback is the mechanism that generally governs homeostatic control. A classic example is the metabolic feedback inhibition in which the activity of the committed-step enzyme in a biosynthetic pathway is inhibited by the final product when that product accumulates above a certain threshold (1–3). Accordingly, the concentration of metabolite oscillates over a narrow range such that the cell immediately compensates for levels below the threshold by increasing synthesis and synthesis is inhibited when the concentration exceeds the threshold. Structural and highly abundant proteins, such as ribosome components and pilin, are also homeostatically regulated by feedback inhibition (4–6). Here we explore the feedback inhibition controlling a structural subunit of the bacterial flagellum (7).

Bacterial flagella are primarily composed of long, helical extracellular polymeric filaments which, when rotated, act like propellers to push cells through the environment (8–10). Each filament is assembled from monomers of the protein flagellin (also known as Hag or FliC) that are synthesized in the cytoplasm and secreted by a dedicated type III secretion system within the flagellar basal body (11–14). Each flagellin monomer is secreted through the lumen of the nascent flagellum and polymerized at the tip of the extending structure by an extracellular chaperone, FliD (15–18). It has been estimated that approximately 20,000 flagellin monomers are needed to synthesize a single flagellar filament of Salmonella enterica, and the metabolic cost of filament synthesis is thought to be substantial (19). Consistent with a high synthetic cost, the flagellin primary sequence has evolved to disfavor energetically expensive amino acids, and complex regulatory pathways restrict flagellin expression (20, 21).

**FIG 1:**
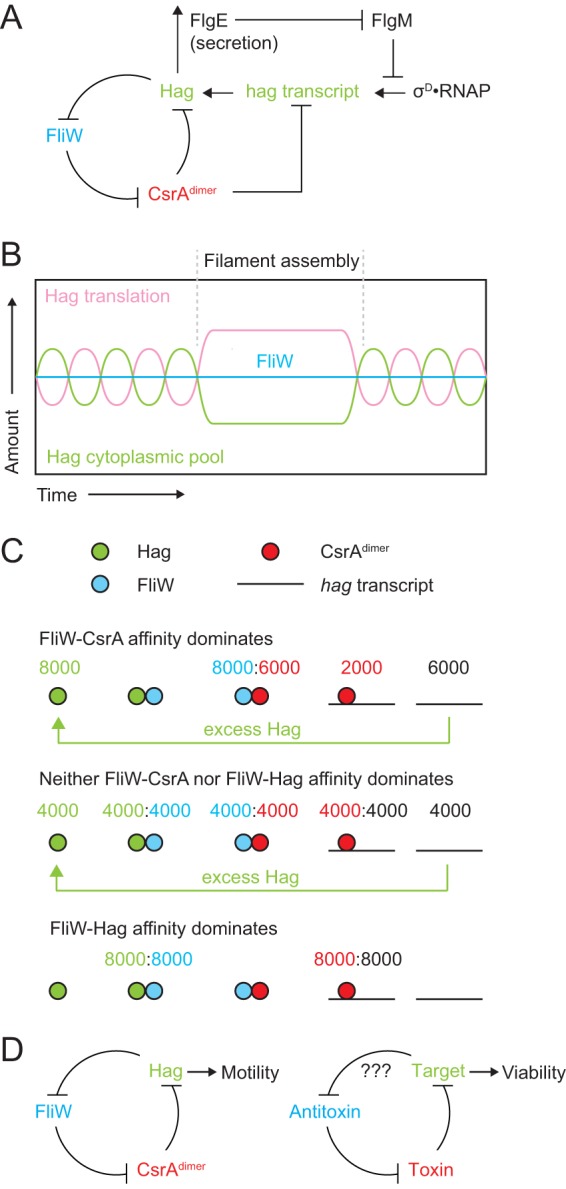
Model of Hag homeostasis. (A) Molecular regulation of the Hag protein and transcriptional and translational levels. T-bars indicate inhibition. Arrows indicate activation. (B) Hag oscillates around the concentration of FliW. Green, relative concentration of Hag in the cytoplasm. Pink, relative level of Hag translation. Blue, concentration of FliW in the cytoplasm. Dashed lines, beginning and end of Hag secretion for filament assembly. (C) Possible interaction regimes of the Hag-FliW-CsrA system. Green, Hag monomers. Blue, FliW monomers. Red, CsrA dimers. Black line, *hag* transcripts. (D) Comparison of the Hag-FliW-CsrA homeostasis module to the toxin-antitoxin module. “???”indicates untested target-antitoxin interaction.

One way in which flagellin expression is restricted is by autoinhibition at the level of translation. In Bacillus subtilis, the Hag flagellin protein autoinhibits its translation through a partner-switching mechanism centered on the proteins FliW and CsrA (7) (Fig. 1A). Cytoplasmic Hag binds and inhibits FliW, and inhibition of FliW is relieved when Hag is exported for polymerization into the filament (22–24). Free FliW then binds and noncompetitively inhibits homodimers of the RNA-binding protein CsrA (CsrA^dimer^), preventing CsrA^dimer^ from binding and occluding the Shine-Dalgarno (SD) sequence of the *hag* transcript to promote Hag translation (25–27). Thus, Hag secretion activates Hag translation for the duration of filament assembly (Fig. 1A and B). Moreover, when filament assembly is complete, secretion is reduced and Hag levels are thought to rise in the cytoplasm, causing Hag to bind to FliW and release CsrA^dimer^, thereby restoring the ability of CsrA to repress *hag* translation (7). *In toto*, Hag-FliW-CsrA constitutes a three-node negative-feedback loop predicted to homeostatically restrict Hag levels within a narrow range of concentrations in the cytoplasm (7, 28). The homeostatic level of Hag relative to that of FliW-CsrA^dimer^ and the biological function for Hag homeostasis are poorly understood.

Here we explore the model of Hag homeostasis by measuring the levels of each protein involved in the negative-feedback loop. We show that low levels of Hag are maintained by equimolar amounts of FliW, CsrA^dimer^, and *hag* transcript such that the system rests at a 1:1:1:1 ratio. The stoichiometric equilibrium was robust with respect to perturbations in flagellar secretion and was ensured by genetic architecture and translational coupling. Thus, Hag feedback is governed by strict stoichiometry poised to increase Hag translation when cytoplasmic Hag levels decrease to below a homeostatic threshold set by the titration of FliW. We further found that the feedback loop is hypersensitive such that even undetectable increases of CsrA levels above equilibrium inhibit motility due to a decrease in *hag* transcript abundance. The Hag-FliW-CsrA system is conserved in the deeper branches of bacterial phylogeny, but the generalized form of structural homeostasis, in which a protein autoinhibits its own synthesis through direct interaction with a simple regulatory module, may be widespread (29–31). We note that the molecular architecture and regulatory logic of FliW-CsrA resemble those of toxin-antitoxin systems (32). Thus, toxin-antitoxin systems could be “homeostasis modules” for essential proteins and limit potentially toxic accumulation of polymerases, ribosomes, or FtsZ.

## RESULTS

### Five percent of the flagellin pool is cytoplasmic.

**FIG 2:**
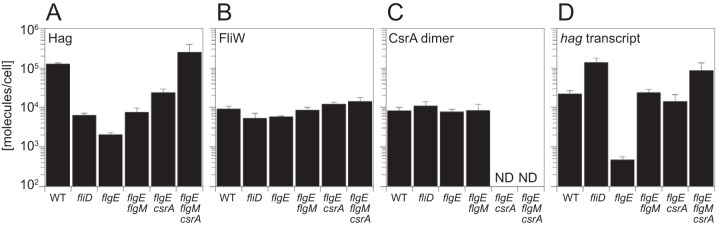
*In vivo* quantification indicates equimolar concentrations of the Hag, FliW, CsrAdimer, and *hag* transcripts. (A to C) Quantitative Western blot analysis of Hag (A), FliW (B), and CsrA_dimer_ (C) using antibodies raised against the Hag, FliW, and CsrA protein, respectively. Standard curves of purified proteins are provided in Fig. S1A, B, and C, respectively. ND, not determined due to the absence of CsrA as a consequence of mutation. (D) Quantitative reverse transcriptase PCR (qRT-PCR) analysis of *hag* transcript. Standard curves of purified *in vitro*-transcribed *hag* transcript are provided in Fig. S1D. The errors bars represent the standard deviations of results from three replicates. The following strains were used to generate all four panels: WT (3610), *fliD* (DS7791), *flgE* (DS4681), *flgE flgM* (DS9622), *flgE csrA* (DS7646), and *flgE flgM csrA* (DS9757).

The flagellar filament is assembled from subunits of the Hag flagellin protein. Two approaches were taken to estimate the number of flagellin molecules per filament. First, cells encoding a cysteine-modified form of the Hag protein (Hag^T209C^) were fluorescently labeled with a maleimide dye, and the average length of 40 filaments was measured by microscopy to be 7 ± 1 μm (33). Multiplying the average length (7 μm) by the value of 2,174 Hag monomers/μm determined by structural analysis of the B. subtilis filament (34) resulted in a value of ∼15,000 Hag proteins/filament. In parallel, quantitative Western blot analysis was conducted on lysates of exponentially grown B. subtilis using an antibody raised against the Hag protein and an infrared-based Licor detection system. A standard curve was generated with purified Hag protein that was diluted with a cell lysate of a *hag* mutant to maximize transfer similarity to the whole-cell lysates with which it would be compared (see Fig. S1A in the supplemental material). An average of 130,000 Hag molecules was calculated per cell (Fig. 2A; see also Table S1 in the supplemental material). Dividing the quantitative Western blotting-based count (130,000 Hag/cell) by an estimated 15 filaments/cell results in a value of approximately 9,000 Hag proteins/filament (35, 36). Averaging the two approaches, we estimated that each filament has ∼12,000 Hag subunits. We conclude that Hag is highly abundant.

10.1128/mBio.00533-19.1 FIG S1 Standard curves used for quantitative Western blotting and qRT-PCR. (A to C) Plotted standard curves representing results of quantitative Western blot analysis of Hag (A), CsrA (B), and FliW (C), with weights (in nanograms [ng]) indicated on the *x* axis and signal on the *y* axis. (D) Plotted standard curve used for qRT-PCR, with the *C_T_* value indicated on the *x* axis and the number of *hag* transcripts on the *y* axis. Blue dots represent the standard curve generated when the *in vitro*-transcribed *hag* transcript was mixed at a 1:1 ratio with RNA extracted from the *hag* deletion background (DS1677). Red dots represent the standard curve generated when the *in vitro*-transcribed *hag* transcript was added to the lysed *hag* deletion background and reextracted (see Materials and Methods for more detail). Download FIG S1, PDF file, 0.1 MB. Copyright © 2019 Oshiro et al. 2019 Oshiro et al. This content is distributed under the terms of the Creative Commons Attribution 4.0 International license.

10.1128/mBio.00533-19.7 TABLE S1 Quantification of Hag, FliW, CsrA, and the *hag* transcript per cell. Download Table S1, DOCX file, 0.04 MB. Copyright © 2019 Oshiro et al. 2019 Oshiro et al. This content is distributed under the terms of the Creative Commons Attribution 4.0 International license.

The Hag protein is found in two types of cellular locations: extracytoplasmic locations (as a polymerized flagellar filament) and cytoplasmic locations (as a soluble pool). To determine the number of Hag molecules in the cytoplasm, extracytoplasmic polymerization was abolished by two different approaches. In one approach, secretion of Hag was abolished by disrupting the *flgE* gene encoding the flagellar hook protein FlgE (37, 38). In another approach, secretion of Hag was made constitutive but polymerization was abolished by disrupting the *fliD* gene encoding the filament cap chaperone FliD (23, 39). Quantitative Western analysis indicated that there were ∼2,000 Hag monomers per *flgE* mutant cell and ∼6,000 Hag monomers per *fliD* mutant cell (Fig. 2A; see also Table S1). We suspect that the cytoplasmic concentration of Hag in the wild-type (WT) strain is probably closer to that observed in the *fliD* mutant as motile cells would need to continuously synthesize new flagella during growth. Thus, by dividing the value of 6,000 monomers in the *fliD* cytoplasm by the value of 130,000 monomers in whole-cell lysates of the wild type, we estimated that the cytoplasmic pool of Hag represents ∼5% of the total.

We speculate that the amount of Hag in the cytoplasm of the *flgE* mutant is lower than that in the theoretical pool in the wild type because Hag transcription and translation are coupled to Hag secretion. When cells cannot complete the flagellar hook (FlgE), the anti-sigma factor FlgM inhibits σ^D^-dependent transcription of the *hag* gene, and *hag* expression is further inhibited at the translational level by the RNA-binding protein CsrA (Fig. 1A) (28, 40–43). To determine the relative contributions of the regulators to the intracellular levels of Hag, the genes *flgM* and *csrA*, encoding FlgM and CsrA, respectively, were mutated in a background that was also mutated for *flgE*. The *flgE flgM* double mutant had ∼8,000 Hag monomers per cytoplasm, likely restricted by CsrA. The *flgE csrA* double mutant had ∼24,000 Hag monomers per cytoplasm, despite the fact that transcription was impaired by FlgM (Fig. 2A; see also Table S1). A *flgE flgM csrA* triple mutant had ∼250,000 Hag monomers, a value greater than that seen with either mutant alone and on par with the total number of subunits observed in the wild type, with the exception that they were entirely trapped within the cytoplasm. We conclude that FlgM and CsrA synergize to prevent cytoplasmic accumulation of Hag and that CsrA is the dominant inhibitor.

### CsrA^dimer^ and FliW are present at a 1:1 ratio in the cytoplasm.

CsrA is part of a three-node negative-feedback loop that restricts Hag translation in a manner dependent on the level of cytoplasmic Hag (7, 28) (Fig. 1A). CsrA binds RNA as a dimer and CsrA^dimer^ proteins are noncompetitively inhibited when bound by FliW (25, 26, 44, 45). FliW switches binding partners between CsrA and Hag to sense Hag levels and to regulate Hag translation accordingly. As the model runs on protein-protein interactions, the relative numbers of those proteins should be important. To determine the amount of FliW and CsrA in the cytoplasm, quantitative Western blot analysis was conducted. Wild-type cells had ∼8,000 CsrA^dimers^ and ∼9,000 FliW monomers in the cytoplasm, representing a nearly equimolar ratio (Fig. 2B and C; see also Table S1). Furthermore, the levels of CsrA^dimer^ and FliW were robust with respect to perturbation as their cytoplasmic concentrations remained relatively unchanged even when flagellar assembly was disrupted by mutation of *flgE* or *flgE* and *flgM* or *fliD*. We infer that Hag levels in the cytoplasm are restricted by the equilibrium concentrations of CsrA^dimer^ and FliW monomers such that the entire system is homeostatically maintained near a 1:1:1 ratio.

Structural analysis indicated that each CsrA^dimer^ was bound by two separate molecules of FliW (FliW_2_:CsrA^dimer^), but the quantitation described above suggested that FliW levels *in vivo* were subsaturating (FliW_1_:CsrA^dimer^) (27). To determine whether one molecule of FliW was sufficient to antagonize a dimer of CsrA, we purified CsrA as a heterodimer (CsrA^Hetero^) in which one monomer was proficient (CsrA^WT^-His_6_) and one monomer was deficient (CsrA^N55D^-strep [Strep-tag]) in binding FliW (26). To generate CsrA^Hetero^, both the CsrA^WT^-His_6_ and CsrA^N55D^-strep fusions were expressed from the same vector and purified using sequential affinity purifications. The heteromeric complex was confirmed by the presence of two bands of similar intensities by the use of sodium dodecyl sulfate-polyacrylamide gel electrophoresis (SDS-PAGE) (Fig. S2). To determine the RNA-binding affinity of CsrA^Hetero^ to the 5′ untranslated region (5′ UTR) of the *hag* transcript (+1 to +100), an RNA electrophoretic mobility shift assay (EMSA) was performed. CsrA^Hetero^ was found to bind the *hag* 5′ UTR with an apparent dissociation constant (*K*_*d*, apparent_ [*K*_*d*, app_]) of 460 nM, a value that was intermediate between the values determined for the CsrA^WT^-His_6_ homodimer (*K_d, app_* = 290 nM) and the CsrA^N55D^-strep homodimer (*K*_*d*, app_ = 960 nM) (Fig. S3A, B, and C). We conclude that CsrA^Hetero^ was functional for RNA binding.

10.1128/mBio.00533-19.2 FIG S2 Purified CsrA constructs. A denaturing gel of purified CsrA constructs loaded in equimolar concentrations and stained with Coomassie brilliant blue is shown. The ratio of the two constructs, CsrA^WT^-His_6_ and CsrA^N55D^-strep, that made up CsrA^(Hetero)dimer^ was determined by densitometry and was found to be 0.88:1. Download FIG S2, PDF file, 0.1 MB. Copyright © 2019 Oshiro et al. 2019 Oshiro et al. This content is distributed under the terms of the Creative Commons Attribution 4.0 International license.

10.1128/mBio.00533-19.3 FIG S3 Binding affinity of CsrA constructs and FliW to the *hag* transcript. (A to D, top) RNA electrophoretic mobility shift assays were performed using the +1 to +100 region of the *hag* transcript and the indicated amounts of CsrA^WT^-His_6_, CsrA^N55D^-strep, CsrA^Hetero(dimer)^, and FliW. “Free RNA” indicates the position of the unbound probe (open triangles). (A to D, bottom) Calculated binding curves and *K*_*d*, apparent_ (*K*_*d*, app_) values for each construct listed above. *K*_*d*, app_ values and standard deviations were calculated with at least three replicates. Download FIG S3, PDF file, 1.1 MB. Copyright © 2019 Oshiro et al. 2019 Oshiro et al. This content is distributed under the terms of the Creative Commons Attribution 4.0 International license.

**FIG 3:**
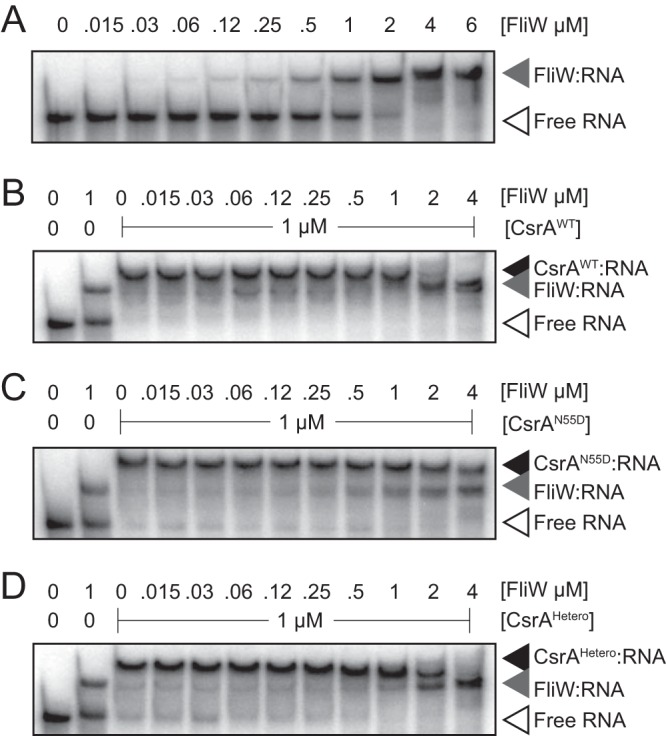
One molecule of FliW is necessary and sufficient to inhibit a dimer of CsrA. (A to D) RNA electrophoretic mobility shift assays (RNA EMSAs) performed using the +1 to +100 region of the *hag* transcript and the indicated amounts of FliW alone (A) or in the presence of CsrA^WT^-His_6_ (B), CsrA^N55D^-strep (C), or CsrA^Hetero(dimer)^ (D). “Free RNA” indicates the position of the unbound probe (open triangles). Black triangles indicate the position of the probe bound by CsrA. Gray triangles indicate the position of the probe bound by FliW. A narrower concentration range of FliW for CsrA inhibition can be seen in Fig. S4. Each gel represents results of experiments repeated in triplicate.

We next sought to determine whether the addition of the FliW protein would antagonize the RNA-binding activity of CsrA^Hetero^, which has only one FliW binding site. As shown previously, addition of FliW alone resulted in an electrophoretic mobility shift of the *hag* 5′ UTR, but the FliW-mRNA affinity was 500-fold weaker than the affinity of FliW for CsrA (7) (Fig. 3A; see also Fig. S3D). Addition of FliW reduced the amount of CsrA^WT^ bound to RNA under conditions of roughly equimolar concentrations, and excess FliW was bound as FliW levels further increased (Fig. 3B; see also Fig. S4A). In contrast, addition of FliW did not appear to decrease the abundance of CsrA^N55D^-bound RNA under conditions of roughly equimolar concentrations but rather appeared to compete for RNA binding at higher concentrations (Fig. 3A and C; see also Fig. S4B). Finally, the shift pattern seen after addition of FliW to CsrA^hetero^ resembled the shift pattern observed with the wild type and was indicative of inhibition (Fig. 3D; see also Fig. S4C). We conclude that while each dimer of CsrA has the capacity to bind two molecules of FliW, the presence of only one molecule of FliW is necessary and sufficient to inhibit the interaction between CsrA^dimer^ and RNA.

10.1128/mBio.00533-19.4 FIG S4 One molecule of FliW is necessary and sufficient to inhibit a dimer of CsrA. (A to C) RNA electrophoretic mobility shift assays (RNA EMSAs) performed using the +1 to +100 region of the *hag* transcript and the indicated amounts of FliW in the presence of CsrA^WT^-His_6_ (A), CsrA^N55D^-strep (B), or CsrA^Hetero(dimer)^ (C). “Free RNA” indicates the position of the unbound probe (open triangles). Black triangles indicate the position of the probe bound by CsrA. Gray triangles indicate the position of the probe bound by FliW. Each gel represents results of experiments repeated in triplicate. Download FIG S4, PDF file, 0.1 MB. Copyright © 2019 Oshiro et al. 2019 Oshiro et al. This content is distributed under the terms of the Creative Commons Attribution 4.0 International license.

**FIG 4:**
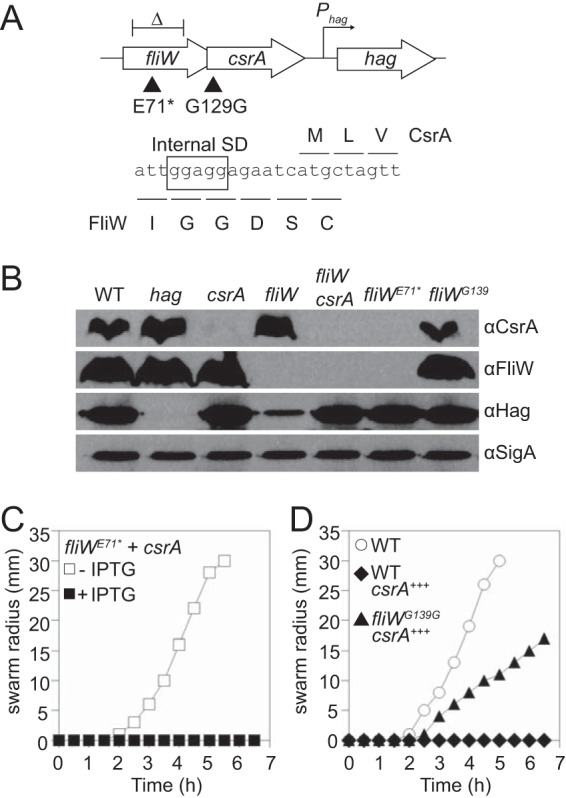
FliW and CsrA stoichiometry is governed by translational coupling. (A) Schematic of the genetic architecture of the *fliWcsrA* region (not drawn to scale). The delta symbol above “*fliW”* indicates the approximate region of the *fliW* inframe markerless deletion. Black triangles indicate the location of the *fliW* premature stop mutation (fliW^E71^^*^) (left) and the *csrA* Shine-Dalgarno mutation (*fliW^G129G^*) (right). (B) Qualitative Western blot analysis of B. subtilis cell lysates probed separately with primary antibodies raised against Hag, FliW, CsrA, and SigA. SigA is used as a loading control. The following strains were used to generate the panel: WT (3610), *hag* (DS1677), *csrA* (DS6188), *fliW* (DS9178), *fliWcsrA* (DK2665), fliW^E71^^*^ (DK4659), and *fliW^G139G^* (DK4660). (C and D) Quantitative swarm expansion assays. The following strains were used to generate the panels: fliW^E71^^*^ *P_hyspank_-csrA* (DK5224 [with IPTG, open square; without IPTG, filled square]), WT (3610 [circles]), *P_hyspank_-csrA* (DS4940 [diamonds]), and *fliW^G139G^ P_hyspank_-csrA* (DK5255 [triangles]). Each point represents the average of results from three replicates. “CsrA^+++^” indicates that CsrA was induced throughout growth and swarming by the addition of 1 mM IPTG.

The amounts of FliW and CsrA^dimer^ appear to be kept at a precise stoichiometric ratio. One way in which the stoichiometric ratio might be maintained is by translational coupling as the reading frame of *fliW* is upstream of and overlaps the reading frame of *csrA* (Fig. 4A). To determine whether translation of CsrA was coupled to that of FliW, a nonsense mutation was introduced into the middle of the *fliW* reading frame to create a premature stop codon (FliW^E71^*). Consistent with translational coupling, the premature stop codon in *fliW* abolished accumulation of both FliW and CsrA, resulting in a resemblance to a *fliW csrA* double deletion mutant (Fig. 4B). That the CsrA levels in the fliW^E71^* allele were undetectable was not a consequence of proteolysis in the absence of FliW, as CsrA was abundant in a strain containing an in-frame markerless deletion of *fliW* that preserved coupling (Fig. 4B). Moreover, overexpression of CsrA alone from an ectopic locus in the fliW^E71^* mutant resulted in motility inhibition, indicating that CsrA expression does not specifically require the presence of FliW (Fig. 4C). We conclude that expression of CsrA is translationally coupled to that of FliW.

Translational coupling often involves internal Shine-Dalgarno sequences, and *csrA* is preceded by what appears to be a separate putative SD sequence (Fig. 4A). To determine the contribution of the *csrA* SD sequence, the sequence was mutated away from the consensus (GGAGG > GGTGG), simultaneously introducing a silent mutation in *fliW* (*fliW^G139G^*; Fig. 4A). The *csrA* SD mutant (*fliW^G139G^*) appeared to result in a slight qualitative reduction in the amount of CsrA protein in Western blot analysis, while FliW protein levels appeared to be unaffected (Fig. 4B). While the effect of the CsrA SD sequence mutation appeared minor, slight changes in the level of CsrA might have phenotypic consequences. To determine the biological consequence of a minor reduction in CsrA levels, CsrA was artificially expressed from an ectopic site in various mutant backgrounds. As previously reported, artificial ectopic expression of CsrA inhibited swarming motility when the native copy of *csrA* was also present in merodiploid (26) (Fig. 4D; see also Fig. S5**)**. Artificial ectopic expression of *csrA* in the *csrA* SD sequence mutant (*fliW^G139G^*) background resulted in only partial inhibition of swarming motility, suggesting that wild-type levels of CsrA from the native site were necessary for full inhibition (Fig. 4D). We conclude that the SD sequence in front of *csrA* plays a seemingly minor but nonetheless important role in maintaining CsrA levels, and we infer that the ratio of FliW to CsrA^dimer^ must be precisely maintained.

10.1128/mBio.00533-19.5 FIG S5 Induction of CsrA at an ectopic site inhibits swarming motility when the wild-type copy of *csrA* is present. Quantitative swarm expansion assay of strains *P_hyspank_-csrA* (DS4940 [left]) and *csrA P_hyspank_-csrA* (DK1522 [right]) was performed under conditions of various concentrations of IPTG induction. Each point represents the average of results from three replicates. Download FIG S5, PDF file, 0.1 MB. Copyright © 2019 Oshiro et al. 2019 Oshiro et al. This content is distributed under the terms of the Creative Commons Attribution 4.0 International license.

**FIG 5:**
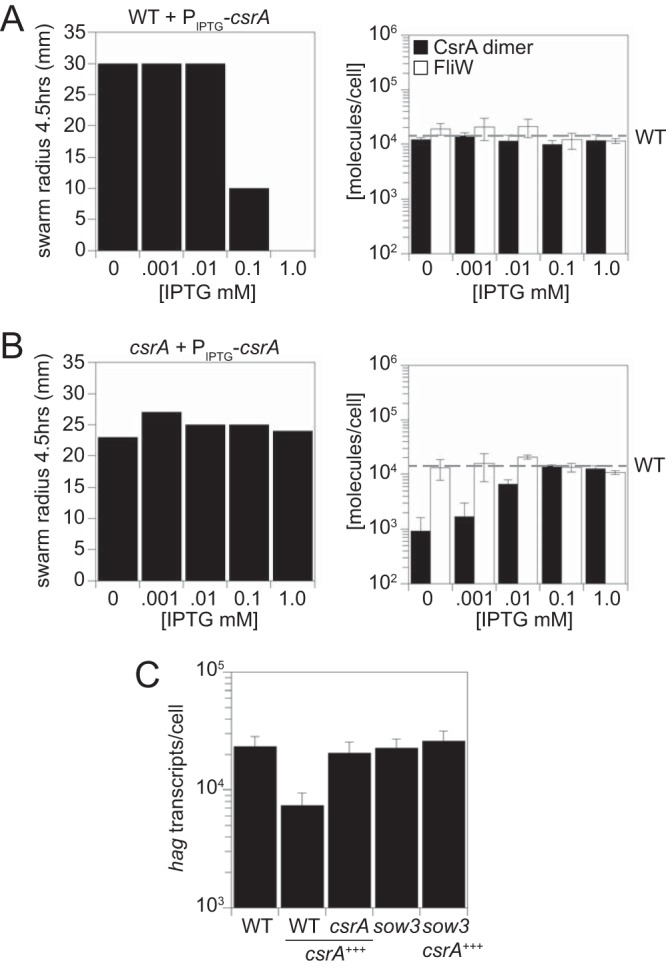
Ectopic expression of CsrA in merodiploid inhibits motility by reducing the amount of *hag* transcript. (A) (Left) Quantitative swarm expansion assay at various concentrations of IPTG present at the 4.5-h time point for strain DS4940 (*P_hyspank_-csrA*). (Right) Quantitative Western blot analysis of amounts of CsrA^dimer^ and FliW at various concentrations of IPTG for the same strain as described above. Error bars represent the standard deviations of results from three replicates. The horizontal dashed bar indicates the level of CsrA^dimer^ in the wild-type strain described in the Fig. 2C legend. (B) (Left) Quantitative swarm expansion assay at various concentrations of IPTG present at the 4.5-h time point for strain DK1522 (*csrA P_hyspank_-csrA*). (Right) quantitative Western blot analysis of the amounts of CsrA^dimer^ and FliW at various concentrations of IPTG for the same strain as described above. Error bars represent the standard deviations of results from three replicates. The horizontal dashed bar indicates the level of CsrA^dimer^ in the wild-type strain described in the Fig. 2C legend. (C) Quantification of the *hag* transcript in the WT (3610), *P_hyspank_-csrA* (DS4940), *csrA P_hyspank_-csrA* (DK1522), *sow3* (DK6073), and *sow3 P_hyspank_-csrA* (DK6082) strains. “*csrA*^+++^” indicates induction by growth in the presence of 1 mM IPTG. Error bars represent the standard deviations of results from three replicates.

To further explore the importance of FliW:CsrA^dimer^ stoichiometry, we titrated CsrA by gradual induction of an ectopically integrated, IPTG (isopropyl-β-d-thiogalactopyranoside)-inducible *csrA* construct in merodiploid with the native copy. Swarming motility was partially inhibited when the construct was induced with 0.1 mM IPTG and was fully inhibited at 1 mM IPTG as previously reported (26) (Fig. 5A, left; see also Fig. S5, left). Quantitative Western blot analysis showed, however, that the levels of CsrA^dimer^ and FliW appeared constant at all levels of IPTG induction, whether or not motility was inhibited (Fig. 5A, right; see also Table S2). We confirmed that the titers of the IPTG-inducible promoter were nonetheless determinable, as CsrA levels increased with increasing amounts of IPTG in a background in which the native copy of *csrA* was deleted (Fig. 5B, right; see also Table S2). In the native *csrA* deletion, maximal IPTG induction elevated CsrA to wild-type levels, but, as previously reported, motility was not inhibited perhaps because native FliW is sufficient (26) (Fig. 5B, left; see also Fig. S5 right). We conclude that maintenance of both the levels and the ratio of FliW to CsrA^dimer^ is robust with respect to artificial perturbation and appears to be restricted to a maximum. We further conclude that motility inhibition from ectopic CsrA expression was not correlated with either an increase in the total complex or an excess of CsrA^dimer^.

10.1128/mBio.00533-19.8 TABLE S2 Quantification of the titration of CsrA dimers and FliW proteins per cell. Download Table S2, DOCX file, 0.1 MB. Copyright © 2019 Oshiro et al. 2019 Oshiro et al. This content is distributed under the terms of the Creative Commons Attribution 4.0 International license.

One way to explain the need for the native copy of *csrA* to inhibit motility when *csrA* was artificially expressed from an ectopic locus is that the location of the native copy was critical and somehow inhibited *hag* translation in *cis*. A *cis* effect is plausible, as the genes encoding CsrA and Hag are adjacently positioned at the native locus. To test the relevance of gene location, the *hag* gene was deleted at the native site and complemented at the ectopic *thrC* site in the chromosome (*thrC*::*P_hag_-hag*). Artificial ectopic expression of *csrA* from the *amyE* site in the chromosome resulted in inhibition of the strain containing the *csrA* gene at the native locus even when the *hag* gene was separated by relocation to *thrC* (Fig. S6). Thus, we conclude that genetic adjacency of *csrA* and *hag* was irrelevant for inhibition and that translation inhibition by CsrA was not due to a *cis* effect. We further conclude that ectopic expression of CsrA inhibits motility in *trans*, likely by titrating levels of FliW expressed from the native locus. Moreover, inhibition is not due to detectable changes in CsrA protein levels and we infer that inhibition is accomplished by another, as-yet-untested variable.

10.1128/mBio.00533-19.6 FIG S6 Inhibition of swarming motility by ectopic expression of CsrA is not due to a *cis* effect. Quantitative swarm expansion assay of strains *hag* (DS1677 [squares]), *hag P_hag_-hag* (DS6235 [circles]), and *hag P_hag_-hag P_hypsank_-csrA* (DK7072 [open diamonds]) was performed. Each point represents the average of results from three replicates. “CsrA^+++^” indicates that CsrA was induced throughout growth and swarming by addition of 1mM IPTG. Download FIG S6, PDF file, 0.1 MB. Copyright © 2019 Oshiro et al. 2019 Oshiro et al. This content is distributed under the terms of the Creative Commons Attribution 4.0 International license.

### *hag* transcript levels are at nearly stoichiometric levels with CsrA^dimer^ proteins.

One variable that could account for the motility inhibition seen under conditions of ectopic expression of CsrA in merodiploid is the stoichiometric ratio between the *hag* transcript and CsrA^dimer^ (7, 25) (Fig. 1A). To measure the number of *hag* transcripts per cell, quantitative reverse transcriptase PCR (qRT-PCR) was conducted. To generate a standard curve that would account for extraction efficiency, defined amounts of *hag* transcript were added to a lysate of a *hag* mutant, reextracted, serially diluted, and used as a template for qRT-PCR (Fig. S1D). Artificial expression of CsrA in merodiploid reduced the levels of *hag* transcripts 3-fold relative to the wild-type levels, and the *hag* transcript levels were restored when *csrA* was artificially expressed in a strain deleted for the native copy of *csrA* (Fig. 5C; see also Table S3). We conclude that artificial expression of CsrA in merodiploid inhibited motility, not because of an increase in total CsrA^dimers^ but rather because of a decrease in the abundance of the *hag* transcript target (Fig. 5A).

10.1128/mBio.00533-19.9 TABLE S3 Quantification of the *hag* transcript under CsrA overexpression conditions. Download Table S3, DOCX file, 0.04 MB. Copyright © 2019 Oshiro et al. 2019 Oshiro et al. This content is distributed under the terms of the Creative Commons Attribution 4.0 International license.

To explore whether a single copy of CsrA encoded at the native site could restrict *hag* transcript levels, qRT-PCR was used to count *hag* transcripts in a variety of mutant backgrounds. Wild-type cells had ∼22,000 copies of the *hag* transcript, representing a slight, 2-to-3-fold excess of the number of CsrA^dimers^ and FliW monomers, which was perhaps consistent with active flagellar filament assembly (Fig. 2D; see also Table S1). When flagellar filament assembly was disrupted by mutation of FliD or FlgE, the levels of *hag* transcripts increased to ∼140,000 copies or decreased to ∼500 copies, respectively (Fig. 2D; see also Table S1). The dramatic changes in copy number were likely due to transcriptional regulation, as cells mutated for *fliD* constitutively secrete both flagellin and FlgM whereas cells mutated for FlgE constitutively accumulate FlgM in the cytoplasm (43). Consistent with transcriptional inhibition by FlgM, mutation of *flgM* in the *flgE* mutant background increased *hag* transcript numbers 48-fold, but *hag* transcript levels did not rise to those observed in the *fliD* mutant (Fig. 2D; see also Table S1). CsrA also appeared to inhibit *hag* transcript levels as transcript numbers increased 28-fold in the *flgE csrA* double mutant and 174-fold in the *flgE flgM csrA* triple mutant to phenocopy the constitutively secreting *fliD* mutant state (Fig. 2D; see also Table S1). Thus, FlgM and CsrA synergize to repress *hag* transcript accumulation and we conclude that CsrA inhibits *hag* transcript accumulation by a mechanism independent of, and in parallel to, that of FlgM.

CsrA is unlikely to inhibit transcription initiation of *hag*, as mutation of *csrA* was shown to have no effect on a *P_hag_* transcriptional reporter construct lacking the CsrA binding site (7). CsrA could inhibit *hag* transcript levels, however, by a mechanism dependent on its RNA binding activity. To determine if *hag* transcript reduction was dependent on direct interaction with CsrA, a *sow3* allele which contains a mutation in the high-affinity binding site for CsrA and reduces the CsrA binding affinity 25-fold was introduced (7). The *sow3* background had amounts of *hag* transcript equivalent to those seen with the wild type and prevented the reduction in *hag* transcript abundance when CsrA was artificially expressed in merodiploid (Fig. 5C; see also Table S3). We conclude that binding of CsrA to the *hag* 5′ untranslated leader sequence both inhibits *hag* translation and restricts *hag* transcript accumulation to maintain nearly equivalent stoichiometries of *hag* transcript, CsrA^dimer^, FliW, and, ultimately, Hag in the cytoplasm.

### Hag homeostasis preserves cellular spatial organization.

**FIG 6:**
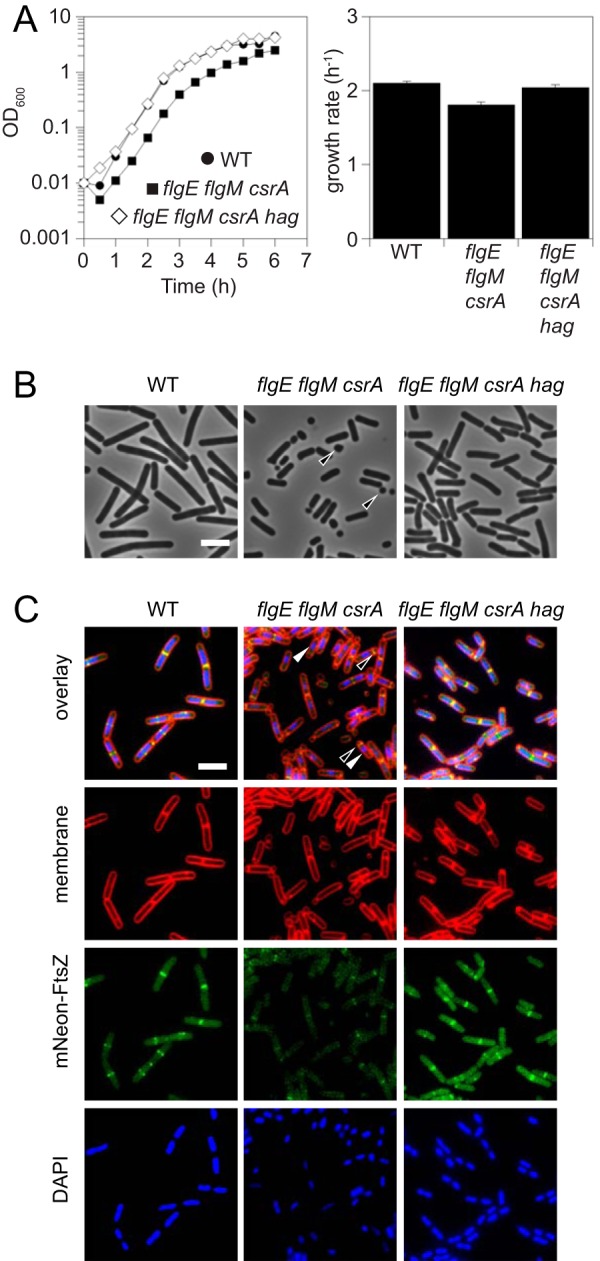
Hag cytoplasmic hyperaccumulation causes cell morphology defects. (A) (Left) Growth curve of WT (3610), *flgE flgM csrA* (DS9757), and *flgE flgM csrA hag* (DK4476) strains. Each point represents the average of results from three replicates. (Right) Growth rates calculated from data shown at the left. Error bars represent the standard deviations of results from three replicates. (B) Phase-contrast micrographs of the strains described in the panel A legend. Open arrowheads indicate large “minicells.” Bar, 4 μm. (C) Fluorescence micrographs of *mNeonGreen-ftsZ* (DK5092), *flgE flgM csrA mNeonGreen-ftsZ* (DK6084), and *flgE flgM csrA hag mNeonGreen-ftsZ* (DK6083). Membranes are stained with FM-4-64 (false red coloring), FtsZ (false green coloring), and nucleoids are stained with DAPI (false blue coloring). Open arrowheads indicate mispositioned FtsZ. Closed arrowheads indicate compacted and asymmetrically positioned nucleoids. Bar, 4 μm.

FlgM and CsrA synergize to restrict the cytoplasmic level of Hag protein, but the physiological reason that Hag levels are tightly restricted is unclear. Compared to the wild type, the *flgE flgM csrA* triple mutant suffered minor growth defects, including a modest increase in lag period, a decrease in growth rate, and a decrease in growth yield (Fig. 6A). The growth-related defects were primarily due to expression of the Hag protein, as a wild-type growth profile was restored by mutating the *hag* gene in the *flgE flgM csrA* triple mutant background (Fig. 6A). Phase-contrast microscopy indicated, however, that the *flgE flgM csrA* triple mutant produced what appeared to be minicells at high frequency and that the minicell production was Hag dependent (Fig. 6B).

Minicells are anucleoid products of aberrant FtsZ ring placement (46–48). To test whether FtsZ rings were mislocalized, a functional mNeonGreen-FtsZ fusion was integrated into various genetic backgrounds (65). Consistent with minicell formation, FtsZ placement in the *flgE flgM csrA* mutant was often polar and the small division products were found to lack nucleoids under conditions of staining with the fluorescent dye DAPI (4′,6-diamidino-2-phenylindole) (Fig. 6C). Moreover, FtsZ fluorescence intensity was reduced in the *flgE flgM csrA* triple mutant, perhaps due to frequent loss of the FtsZ pool to the inert minicell population. Finally, nucleoids of the *flgE flgM csrA* triple mutant appeared to be highly compacted and often uncentered in the cytoplasm (Fig. 6C). The cytological defects observed in the *flgE flgM csrA* triple mutant were corrected when *hag* was also deleted (Fig. 6C). We conclude that hyperaccumulation of Hag in the cytoplasm caused generalized defects in intracellular organization and that Hag homeostatic control is necessary to ensure proper cell growth and division in motile cells.

## DISCUSSION

The bacterial flagellum is an elaborate multisubunit complex assembled from dozens of different structural proteins in numbers that range from 6 copies of a particular rod subunit to the ∼12,000 copies of flagellin measured here (34, 49). How the relative stoichiometries of flagellar structural subunits are rigorously governed over 4 orders of magnitude is poorly understood. Here we show that Hag levels in the cytoplasm are restricted to approximately 5% of the total pool by a roughly 1:1:1:1 ratio of Hag/FliW/CsrA^dimer^/*hag* transcript at equilibrium, relieved by Hag export during filament assembly (Fig. 1A). Strict equimolar ratios permit hypersensitive detection of Hag by FliW titration and an equally sensitive response in Hag translation as CsrA^dimer^ titrates *hag* transcripts. We predict that the homeostatic level of Hag oscillates in the cytoplasm over a narrow concentration range that is ultimately set by the amount of FliW (Fig. 1B).

FliW binds either one molecule of Hag or one dimer of CsrA in a partner-switching mechanism. If the affinity of the FliW-CsrA interaction dominated over or was even equal to that of Hag *in vivo*, the levels of free *hag* transcript should be abundant (Fig. 1C, top and middle). An abundance of free *hag* transcript would cause Hag levels in the cytoplasm to increase and significantly exceed those of FliW before equilibrium would be restored, but that was not observed. Instead, we found that Hag and FliW levels were nearly equivalent *in vivo*, and thus we infer that the Hag-FliW interaction likely dominates (Fig. 1C, bottom). If FliW is primarily sequestered by Hag, then CsrA^dimer^ proteins primarily bind to and inhibit translation of *hag* transcripts (Fig. 1C bottom). Domination of the FliW-Hag interaction would be advantageous to permit detection of nearly single-molecule variations in the Hag protein pool; for every molecule of Hag by which the amount of FliW exceeds that of Hag, one molecule of *hag* transcript is released to support compensatory translation (Fig. 1B). Seemingly inconsistent with a dominating FliW-Hag interaction is that FliW appears to have a higher affinity for CsrA^dimer^ than Hag *in vitro* (7), but the relative pairwise affinity does not take into account the other members of the *in vivo* system, including the *hag* transcript. Thus, based on the *in vivo* quantitation performed here, we conclude that FliW provides hypersensitive detection of Hag and that Hag oscillates around the FliW concentration at steady state.

Just as one molecule of Hag inhibits one molecule of FliW, here we show that only one molecule of FliW is needed to antagonize a dimer of CsrA. Three-dimensional (3D) structure analysis of the complex clearly indicates, however, that a second molecule of FliW can bind to a CsrA^dimer^, and secondary binding may be a mechanism to dampen the derepression of Hag translation (27). During steady-state Hag oscillation, relatively few FliW molecules are released, and likely each binds to free CsrA dimers. When Hag concentrations drop rapidly, however, perhaps when two or more flagellar basal bodies simultaneously draw from the pool to assemble filaments, an excess of FliW might be released that could bind either to unbound CsrA dimers or to CsrA dimers already bound to one FliW. As a consequence, all of the FliW_2_:CsrA^dimer^ pairings would create equal numbers of CsrA^dimer^ bound transcripts and dampening would occur (Fig. 1C, bottom). The critical 1:2 stoichiometric ratio of FliW to CsrA is governed, at least in part, by genetic architecture and translational coupling such that even small reductions in the level of CsrA, such as those caused by mutation of an internal ribosome binding site (RBS), have biological consequences on motility. We predict that other systems must also be present to restrict FliW-CsrA abundances, as their levels were constant and CsrA could not be artificially forced to hyperaccumulate.

Ectopic transcription of CsrA from a strong artificial promoter inhibited motility only when it was expressed in merodiploid (24); the motility inhibition was not due to CsrA hyperaccumulation and was instead due to a reduction in the level of *hag* transcript. CsrA was found to be inhibitory with respect to *hag* transcript levels in general, as a *csrA* mutation elevated *hag* transcript levels in a *flgE* mutant nearly as much as mutation of the transcription-level anti-sigma factor FlgM. The mechanism by which CsrA restricts *hag* transcript levels is unknown but requires the CsrA binding site within the RNA target, and CsrA may control transcript stability such as it does for some transcripts in other organisms (50–54). The amount of *hag* transcript corresponding to the 5′ UTR is important and is regulated at multiple levels, and we note that FliW also binds the *hag* leader sequence for as-yet-unknown reasons. Ultimately, *hag* transcript can be made to hyperaccumulate either in a strain in which FlgM and CsrA are antagonized by constitutive secretion of flagellin (*fliD*) or in a strain that cannot secrete flagellin and that is simultaneously mutated for both FlgM and CsrA (*flgE flgM csrA*). In the case of the *flgE flgM csrA* triple mutant, derepression of both transcription and translation caused hyperaccumulation of the Hag protein in the cytoplasm such that its levels exceeded the amount normally polymerized on the outer side of the cell.

The biological reason for Hag homeostasis is unknown, but one reason could be a need to limit the energetic expenditure on flagellar assembly. We tend to think that energy conservation is of minor advantage, at least in nutrient-rich environments, as the *flgE flgM csrA* triple mutant produced more flagellin than the wild type but suffered only minor defects in growth. Instead, flagellin hyperaccumulation resulted in cells with severe defects in spatial organization, including, but perhaps not limited to, high-frequency FtsZ-ring misplacement, compacted asymmetrically positioned nucleoids, and aberrant cell morphology. While Hag could bind to and interfere with a specific cell division target, the generality of the defect suggests a nonspecific mechanism. One mechanism by which Hag hyperaccumulation could cause generalized space defects is by an increase in molecular crowding. The volume of a 4-μm-by-1-μm B. subtilis cell is roughly 4 × 10^9^ nm^3^, and a structure-based approximation of the volume of a single Hag subunit is 8.0 × 10^4^ nm^3^ (34). The homeostatic level of 6,000 subunits of Hag occupies approximately 0.06% of the wild-type cytoplasm, but that level increases to 2.5% of the total volume in the hyperaccumulating *flgE flgM csrA* triple mutant. The 62-fold volumetric increase in the level of the Hag pool may be significant in an already crowded cytoplasm, and Hag homeostasis may thus be critical to minimize molecular crowding. While nutrients may or may not be limiting, depending on the environment, we suggest that cytoplasmic volume is always at a premium.

Structural homeostasis may be a common mechanism to restrict hyperaccumulation of enzymes and structural proteins in the cell. While FliW-CsrA is highly specific for flagellin, we note that the genetic and molecular architecture of the module is remarkably similar to that of some type II toxin-antitoxin systems (32) (Fig. 1D). As with the genes encoding FliW-CsrA, toxin-antitoxin genes are often translationally coupled, the stoichiometric ratio of toxin to antitoxin is thought to be critical for function, and antagonism of the protein products is mediated by direct interaction (55–57). Moreover, both systems have mechanisms to restrict their own synthesis, and some toxins have very specific targets (58–60). All that remains to complete the analogy with FliW-CsrA is to have the target of the toxin bind and inhibit antitoxin activity (Fig. 1D). While other binding partners for antitoxins have not been reported, the possibility that other targets might exist may not have been considered (Fig. 1D). Thus, FliW could be considered an “antitoxin,” and CsrA could be considered a “toxin” that happens to act on a nonessential target, in this case, Hag. Conversely, some toxin-antitoxin systems could be homeostasis modules that restrict accumulation of essential proteins that could be harmful when synthesized in excess.

## MATERIALS AND METHODS

### Strains and growth conditions.

B. subtilis strains were grown in lysogeny broth (LB) broth (10 g tryptone, 5 g yeast extract, 5 g NaCl per liter) or on LB plates fortified with 1.5% Bacto agar at 37°C. When appropriate, antibiotics were included at the following concentrations: 10 μg/ml tetracycline (tet), 100 μg/ml spectinomycin (Spec), 5 μg/ml chloramphenicol (Cm), 5 μg/ml kanamycin (Kan), and 1 μg/ml erythromycin plus 25 μg/ml lincomycin (*mls*). Isopropyl β-d-thiogalactopyranoside (IPTG; Sigma) was added to the medium at the indicated concentration when appropriate.

For quantitative swarm expansion assay, strains were grown to mid-log phase (optical density at 600 nm [OD_600_] of 0.3 to 1.0) and concentrated to an OD_600_ of 10 in pH 7.4 phosphate-buffered saline (PBS) (137 mM NaCl, 2.7 mM KCl, 10 mM Na_2_HPO_4_, 2 mM KH_2_PO_4_) containing 0.5% India ink (Higgins). LB plates containing 0.7% Bacto agar with or without various concentrations of IPTG were dried for 10 min in a laminar flow hood, centrally inoculated with 10 μl of the cell suspension, dried for another 10 min, and incubated at 37°C in a humid chamber. The swarm radius was measured along the same axis every 30 min.

### Microscopy.

Fluorescence microscopy was performed with a Nikon 80i microscope with a phase-contrast objective (Nikon Plan Apo; 100×) and an Excite 120 metal halide lamp. FM4-64 dye [*N*-(3-triethylammoniumpropyl)-4-(6-(4-(diethylamino)phenyl)hexatrienyl)pyridinium dibromide] was visualized with a C-FL HYQ Texas Red filter cube (excitation filter wavelength of 532 to 587 nm, barrier filter wavelength of >590 nm). DAPI was visualized using a UV-2E/C DAPI filter cube (excitation filter wavelength of 340 to 380 nm, barrier filter wavelength of 435 to 485 nm). mNeonGreen was visualized using a C-FL HYQ fluorescein isothiocyanate (FITC) filter cube (FITC; excitation filter wavelength of 460 to 500 nm, barrier filter wavelengths of 515–550 nm). Images were captured with a Photometrics Coolsnap HQ2 camera in black and white, subjected to false coloring, and superimposed using Metamorph image software.

To stain membranes and nucleoids, cells were grown at 37°C to an OD_600_ of 0.6 to 1.0, and 1 ml of culture was centrifuged, resuspended in 50 μl PBS containing 5 μg/ml FM4-64 (Molecular Probes) and 1 μg/ml DAPI (Molecular Probes), and incubated at room temperature for 10 min. Samples were observed by spotting 4 μl of suspension on either a microscope slide (followed by immobilization with a poly-l-lysine-treated coverslip) or an agarose pad (which was then covered with a glass coverslip). Agarose pads were creating by making a 1% solution of electrophoresis-grade agarose (Fisher Scientific) mixed in PBS and applying the molten solution to a slide, covering the molten solution with a glass microscope slide, and allowing the molten solution to cool for 5 min prior to use.

For fluorescence microscopy and measurement of flagellar filaments, a strain (DK5687) with a reduced number of flagella and encoding the modified Hag protein Hag^T209C^ was collected as described above but was resuspended in 50 μl of PBS buffer containing 5 μg/ml Alexa Fluor 488 C_5_ maleimide (Molecular Probes), and incubated for 5 min at room temperature (33). Cells were then washed twice with 500 μl PBS buffer and were stained for membranes as described above. Four microliters of suspension was placed on a glass microscope slide and immobilized with a poly-l-lysine-treated coverslip. Approximate filament lengths were measured using the measurement tool of ImageJ (61).

### Strain construction.

All constructs were either introduced into a 3610-derived natural competent strain DK1042 (62) or introduced first by natural competence into a domesticated strain (PY79) or a 3610-derived competent strain cured of pBS32 plasmid DS2569 (62) and then transferred to the 3610 background using bacteriophage SPP1-mediated generalized phage transduction (63). Briefly, SPP1-mediated transduction was performed by generating a lysate on B. subtilis grown in TY (1% tryptone, 0.5% yeast extract, 0.5% NaCl, 10 mM MgSO_4_, 1 mM MnSO_4_). Recipient strains were grown to the stationary phase in TY, a 1-ml volume was diluted into 9 ml TY, and 25-μl volumes (for streptomycin) of lysates were added, following by incubation at room temperature for 30 min and selection for the respective antibiotic at 37°C overnight. For transductions in which spectinomycin resistance was selected for, 10 mM sodium citrate was added to the selection plate. All strains used in this study are listed in Table 1. All primers and plasmids used in this study are listed in Table S4 in the supplemental material.

**TABLE 1:** Strains

Strain	Genotype
3610	Wild type
DK1042	*comI^Q12L^* (62)
DK1522	*ΔcsrA amyE*::*P_hyspank_-csrA spec* (26)
DK2665	*ΔfliWcsrA comI^Q12L^*
DK4205	*comI^Q12L^ csrA^N55D^*
DK4476	*comI^Q12L^ ΔcsrAhag flgM*::*tet ΔflgE*
DK4659	fliW^E71^* *comI^Q12L^*
DK4660	*fliW^G139G^ comI^Q12L^*
DK5092	*mNeonGreen-ftsZ comI^Q12L^*
DK5224	fliW^E71^* *amyE*::*P_hyspank_-csrA spec comI^Q12L^*
DK5225	*fliW^G139G^ amyE*::*P_hyspank_-csrA spec comI^Q12L^*
DK5687	*Δhag amyE*::*P_hag_-hag^T209C^ spec swrA*::*tet*
DK6073	*hag^sow3^ comI^Q12L^*
DK6082	*hag^sow3^ amyE*::*P_hyspank_-csrA spec comI^Q12L^*
DK6083	*flgM*::*tet ΔcsrAhag ΔflgE mNeonGreen-ftsZ comI^Q12L^*
DK6084	*ΔflgE flgM*::*tet csrA*::*kan mNeonGreen-ftsZ comI^Q12L^*
DK7072	*Δhag thrC*::*P_hag_-hag^T209C^ mls amyE*::*P_hyspank_-csrA spec*
DS1677	*Δhag* (33)
DS2569	*ΔpBS32* (62)
DS4681	*ΔflgE* (38)
DS4940	*amyE*::*P_hyspank_-csrA* (7)
DS6188	*ΔcsrA* (7)
DS7646	*ΔflgE ΔcsrA*
DS7791	*ΔfliD* (23)
DS9178	*ΔfliW*
DS9622	*ΔflgE flgM*::*tet*
DS9757	*ΔflgE flgM*::*tet csrA*::*kan*

10.1128/mBio.00533-19.10 TABLE S4 Primers and plasmids. Download Table S4, DOCX file, 0.1 MB. Copyright © 2019 Oshiro et al. 2019 Oshiro et al. This content is distributed under the terms of the Creative Commons Attribution 4.0 International license.

### Native site mutant.

Site-directed mutations at the native site were performed by allelic replacement. 3610 genomic DNA was amplified using primer pairs 5434/4973 and 5435/4972 for *csrA^N55D^*, 5434/5353 and 5435/5352 for *fliW^E71stop^*, 5434/5372 and 5435/5371 for *fliW*^(^*^G139G; ggagg-> ggtgg^*^)^, and 5434/6286 and 5435/6285 for *hag^sow3^*. The pairs of fragments were then assembled together by isothermal “Gibson” assembly (64), subjected to restriction digestion using BamHI and KpnI, and introduced into the respective sites of pMiniMAD, which carries a temperature-sensitive origin of replication and an erythromycin resistance cassette, to generate pRO25, pRO30, pRO33, and pRO68. The plasmids were subsequently subjected to passage through *recA*-positive (*recA*+) Escherichia coli strain TG1 and transformed into DK1042. Plasmid pMinimad (and its derivatives pRO25, pRO30, pRO33, and pRO68) encodes an *mls* resistance cassette and a temperature-sensitive origin that is active at room temperature but not at 37°C. Plasmid integration was selected for by *mls* resistance at 37°C. To evict the plasmid, the strain was subsequently incubated at a room temperature overnight without antibiotic selection. Cells were then serially diluted and plated on LB agar at 37°C. Individual colonies were patched onto LB plates containing or not containing *mls* to identify *mls*-sensitive colonies that had evicted the plasmid. Chromosomal DNA from colonies that had excised the plasmid was purified, and an amplicon for sequencing was PCR amplified using primer pair 1541/4641 and sequenced for the appropriate mutation using primers 1541/1544 for pRO25, pRO30, and pRO33 and primers 1873/4641 for pRO68.

### In-frame deletions.

To generate the *ΔfliW* and *ΔfliWcsrA* in-frame markerless deletion constructs in the DK1042 background, plasmid pJP87 and pSG36, respectively, were introduced and excised following the protocol described above. Deletions of *fliW* and *fliWcsrA* were verified by PCR using primers 1871/1907.

To generate strains with a *ΔflgE* in-frame markerless deletion, plasmid pDP306 was introduced and excised following the protocol described above. Deletion of *flgE* was verified by PCR using primers 1483/1486.

**(i) Insertion-deletion alleles.** The *ΔcsrA*::*kan* (7) and *ΔflgM*::*tet* (43) insertion-deletion alleles was generated by long-flanking homology PCR (7, 43) and introduced into strain PY79 by natural competence and further introduced into appropriate strain backgrounds by SPP1-mediated transduction.

**(ii) *ftsZ*::*mNeonGreen*-*ftsZ*.** The *ftsZ*::*mNeonGreen-ftsZ* construct was a gift from Ethan Garner and was introduced following a protocol that was described previously (65). Briefly, *erm-ftsA-mNeonGreen-ftsZ-cat* was introduced into the appropriate strain backgrounds by SPP1-mediated transduction. After integration, plasmid pDR244 (containing Cre recombinase and a temperature-sensitive origin of replication) (a gift from David Rudner) was introduced by SPP1-mediated phage transduction, grown at 30°C, and selected for on Spec. Transductants were struck on LB, LB-Cm, LB-MLS, and LB-Spec and grown at 37°C overnight to enable us to look for colonies that grew only on LB.

**(iii) P_hag_-hag^T209C^.** The P_hag_-hag^T209C^ complementation construct at the ectopic site *lacA* (the T209C mutation is functional and allows labeling of the flagellar filament with cysteine-reactive maleimide dyes) was generated by restriction digestion of plasmid pNE4 with BamHI and SalI to excise P_hag_-hag^T209C^ and was then introduced into the respective restriction sites in pDR183 to generate pKB142. Plasmid pKB142 was introduced into DS2569 by natural competence and was further introduced into appropriate strain backgrounds by SPP1-mediated transduction.

**(iv) *P_hyspank_-csrA*.** The IPTG-inducible construct *P_hyspank_-csrA* was introduced into the ectopic site *amyE* by selection for spectinomycin resistance in appropriate strain backgrounds by SPP1-mediated transduction using a lysate generated from strain DS4940.

**(v) CsrA^N55D^-strep expression vector.** To generate a translational fusion of CsrA^N55D^ to the Strep tag, a fragment containing *csrA*^N55D^ was amplified by using DK4205 as a template and primer pair 6315/6316 and was digested with NcoI/EcoRI. The fragment was ligated into the NcoI and EcoRI sites of pETDUET-1 containing an ampicillin resistance cassette to create pRO77.

**(vi) CsrA^N55D^-strep tag/CsrA^WT^-His_6_ heterodimer expression vector.** To generate a vector that expressed both CsrA^N55D^-strep and CsrA^WT^-His_6_, a fragment containing CsrA-His_6_ was amplified by using pCSB9 as a template and primer pair 4554/5967 and was digested with NdeI/KpnI. The fragment was ligated into the NdeI/KpnI sites of pRO77 to create pRO76.

### CsrA-His_6_ protein purification.

CsrA^WT^-His_6_ expression vector pCSB9 was transformed into BL21(DE3) E. coli and grown to an OD_600_ of ∼0.7 in 500 ml of Terrific broth (900 ml deionized water, 24 g yeast extract, 20 g tryptone, and 4 ml glycerol), induced with 1 mM IPTG, and grown overnight at 16°C. Cells were pelleted and resuspended in CsrA lysis buffer (100 mM Tris-HCl [pH 8.0], 400 mM NaCl), treated with lysozyme, and lysed by sonication. Lysed cells were centrifuged at 14,000 × *g* for 30 min. The cleared supernatant was combined with nickel-nitrilotriacetic acid (Ni-NTA) resin (Novagen) and incubated for 1 h at 4°C. The bead/lysate mixture was poured onto a 1-cm separation column (Bio-Rad), and the resin was allowed to pack and was washed with CsrA wash buffer (50 mM Tris-HCl [pH 8.0], 200 mM NaCl, 10% glycerol). CsrA-His_6_ bound to the resin was then eluted using a stepwise elution of CsrA wash buffer with 5, 15, and 500 mM imidazole. Eluted proteins were separated by SDS/PAGE and subjected to Coomassie staining to verify purification of CsrA-His_6_, and appropriate fractions were pooled and concentrated to ∼2 ml. Final purification of CsrA-His_6_ protein was conducted via size exclusion chromatography on a Superdex 75 16/60 column (GE Healthcare) using CsrA gel filtration buffer (20 mM Tris-HCl [pH 8.0], 200 mM NaCl, 10% glycerol, 1 mM EDTA, pH 8.0), and the fractions were concentrated and stored at −20°C. The concentration of CsrA-His_6_ was determined by Bradford assay (Bio-Rad).

### CsrA^N55D^-strep tag protein purification.

CsrA^N55D^-strep expression vector pRO77 was transformed into Rosetta-gami E. coli, and samples were induced, collected, and lysed following the protocol described above. The cleared supernatant was combined with Strep-Tactin Sepharose (IBA Solutions) and incubated for 1 h at 4°C. The bead/lysate mixture was poured onto a 1-cm separation column (Bio-Rad), and the resin was allowed to pack and was washed with buffer W (IBA Solutions). CsrA^N55D^-strep bound to the resin was then eluted using buffer E (IBA Solutions). Eluted proteins were separated by SDS/PAGE and subjected to Coomassie staining to verify purification of CsrA^N55D^-strep. Final purification, storage, and concentration determination of CsrA^N55D^-strep protein were conducted following the protocol described above.

### CsrA^N55D^-strep/CsrA-His_6_ heterodimer purification.

To purify a CsrA heterodimer (CsrA^N55D^-strep::CsrA-His_6_), CsrA^N55D^-strep and CsrA-His_6_ were expressed simultaneously from dual-expression vector pRO76 by transformation into Rosetta-gami E. coli. The CsrA heterodimer was affinity purified following the His_6_ tag protocol described above. Eluted proteins were separated by SDS/PAGE and stained with Coomassie brilliant blue to verify purification of both CsrA^N55D^-strep and CsrA-His_6_. Fractions containing both constructs were pooled and concentrated to ∼5 ml, and an equal volume of CsrA wash buffer was added to dilute out the imidazole. The pooled fractions were subsequently affinity purified a following the strep-tag protocol described above. Eluted proteins were separated by SDS/PAGE and subjected to Coomassie staining to verify the presence of both CsrA^N55D^-strep and CsrA-His_6_. Final purification, storage, and concentration determination of CsrA Heterodimer were conducted by following the purification protocols described above.

### His_6_-SUMO-FliW and -Hag protein purification.

SUMO-FliW protein expression vector pSM12 and SUMO-Hag protein expression vector pSM56 were transformed into Rosetta-gami E. coli. Purification of the SUMO-FliW protein was performed by following the purification protocol described above for CsrA-His_6._ Purification of SUMO-Hag protein followed a protocol similar to that described above for CsrA-His_6_ but differed with respect to the buffer conditions by the use of the following buffers: lysis buffer (50 mM Na_2_HPO_4_, 300 mM NaCl, 10 mM imidazole), wash buffer (50 mM Na_2_HPO_4_, 300 mM NaCl, 30 mM imidazole), and elution buffer (50 mM Na_2_HPO_4_, 300 mM NaCl, 500 mM imidazole). Eluted proteins were separated by SDS/PAGE and subjected to Coomassie staining to verify purification of the SUMO-FliW/Hag fusion. Purified SUMO-FliW/Hag was combined with ubiquitin ligase (protease) and cleavage buffer and incubated overnight at 4°C to cleave the SUMO tag (66). The cleavage reaction mixture was combined with Ni-NTA beads, incubated for 2 h at 4°C, and centrifuged to pellet the resin. Supernatant for purified Hag protein was dialyzed in 1× PBS (pH 8.0) supplemented with 50% glycerol. Purified FliW protein was further cleaned following the same final purification step as that described for CsrA-His_6_. FliW and Hag were stored at −20°C. Removal of the SUMO tag was verified by SDS/PAGE and Coomassie staining.

### Qualitative Western blotting.

B. subtilis strains were grown in LB to an OD_600_ of ∼1.0, and 10 ml of sample was harvested by centrifugation, resuspended to reach an OD_600_ of 100 in lysis buffer (20 mM Tris [pH 7.0], 10 mM EDTA, 1 mg/ml lysozyme, 10 μg/ml DNase I, 100 μg/ml RNase I, 1 mM phenylmethylsulfonyl fluoride [PMSF]), and incubated for 60 min at 37°C. Cell lysates were diluted 1:10 for samples being probed by anti-Hag and anti-SigA antibodies. A 10-μl volume of lysate was mixed with 2 μl 6× SDS loading dye. Samples were separated by 15% sodium dodecyl sulfate-polyacrylamide gel electrophoresis (SDS-PAGE). The proteins were electroblotted onto nitrocellulose and developed with anti-CsrA (1:5,000) (24), anti-FliW (1:10,000) (24), anti-Hag (1:80,000) (7), or anti-SigA (generous gift of Masaya Fujita, University of Houston) (1:80,000) and a 1:10,000 dilution secondary antibody (horseradish peroxidase [HRP]-conjugated goat anti-rabbit immunoglobulin G). The immunoblot was developed using an Immun-Star HRP developer kit (Bio-Rad).

### Quantitative Western blotting.

A standard curve was generated for each protein using purified Hag, FliW, or CsrA protein. A 2-μl volume of purified protein was mixed with 10 μl of appropriate deletion strain lysate (collected using qualitative Western blotting methods as described above) and mixed with 6× loading dye (1× final concentration). Samples were collected and prepared following the protocol described above but were also serially diluted and plated for CFU counts; testing of each strain was done in triplicate. Cell lysates were diluted 1:10 for the samples probed by anti-Hag, anti-SigA, and anti-EFP antibodies (67) (1:40,000). The standard curves were determined and the samples were separated by 15% SDS-PAGE; each gel had a standard curve. The proteins were electroblotted and developed following the protocol described above except using a 1:20,000-dilution secondary antibody (Alexa Fluor 750-conjugated goat anti-rabbit immunoglobulin G; Life Technologies). Immunoblots were scanned using an Odyssey infrared imager (Li-COR), and signal was measured using Image Studio Lite (version 5.0.21). The signal from each sample was compared to the corresponding standard curve to determine the amount of protein of interest in that sample. The amount of protein was converted to picomole values using Promega Biomath Calculator molar conversions (http://www.promega.com/a/apps/biomath/).

### Calculating levels of protein per cell.

The signal from each sample was compared to the corresponding standard curve to determine the amount of protein of interest in that sample. The amount of protein was converted to picomole values using Promega Biomath Calculator molar conversions (http://www.promega.com/a/apps/biomath/).

The number of cells loaded per lane was calculated by the following equations: (CFU/milliliter) * (sample volume) = CFU; CFU/(volume of lysis buffer in which the cells were resuspended) = CFU/microliter; (CFU/μl) * (microliters of sample loaded/lane) = CFU/lane.

The number of proteins per cell was calculated by the following equation: (moles of protein/lane) × (CFU/lane)^−1^ × (6.02 * 10^23^/mol) = number of proteins/cell.

### RNA electrophoretic mobility shift assay.

The +1 to +100 region relative to the *hag* transcription start site was *in vitro*-transcribed using a PCR-derived template with a T_7_ promoter and internally labeled with [α-^32^P]ATP. The template was generated using 3610 genomic DNA and primer pair 6690/6691, and the template was subjected to phenol-chloroform extraction. The template was incubated with T7 RNA polymerase, nucleoside triphosphates (NTPs) (100 mM [each] CTP, GTP, and UTP; 5 mM ATP; and radioactive [α-^32^P]ATP) (68). Unincorporated nucleotides were removed by the use of a Chroma-spin column (Clontech), and the RNA was further cleaned by gel purification. RNA concentrations were calculated by the use of a scintillation counter. Various concentrations of CsrA proteins were incubated with 20 pM of radiolabeled RNA for 30 min at 37°C in CsrA binding reaction buffer (40 mM Tris-HCl [pH 7.5], 4 mM MgCl_2_, 100 mM KCl, 20 ng/μl yeast RNA [Invitrogen], 20 mM dithiothreitol [DTT], 10% glycerol, 4 U of RNase inhibitor [Promega]) (69). Reactions that contained FliW, CsrA, and the radiolabeled probe were incubated as described above; the FliW protein was then added at various concentrations, and the reaction mixture was incubated for an additional 5 min at 37°C. After incubation, the reaction mixtures were fractionated on native 15% polyacrylamide gels (Bio-Rad) (19:1 acrylamide/bisacrylamide) at room temperature at 24 mA (constant) for 2 to 3 h with Tris-glycine-EDTA running buffer (30 mM Tris base, 190 mM glycine, and 1 mM EDTA, disodium salt). Gels were dried and exposed to phosphorimager screening overnight and were subjected to autoradiography (Typhoon FLA 9500). ImageQuant TL (1D; v8.1) was used to quantify the bound and free RNA. RNA-binding isotherms were obtained using GraphPad Prism 6 with a curve fit of specific binding with Hill slope.

### RNA isolation.

B. subtilis strains were grown in LB with or without 1 mM IPTG, when appropriate, to an OD_600_ of ∼1.0; 20 ml of culture was added to an equal volume of cold methanol (−80°C) and harvested by centrifugation at 5,000 × *g* for 10 min at 4°C; and the supernatant was removed and stored at −80°C (70). For quantitative RT-PCR, cultures were serially diluted onto LB plates to determine CFU levels per milliliter. Pellets were resuspended in 3.2 ml hot LETS buffer (10 mM Tris-HCl [pH 7.4], 50 mM LiCl, 10 mM EDTA [pH 8.0], 1% SDS, 75°C), subsequently added to a mixture of ∼2.6-g acid-washed glass beads (Sigma) and 2.4 ml hot acid-saturated phenol (Fisher Bioreagents) (pH 4.6, 75°C), and subjected to vortex mixing for 3 min. Chloroform (Macron Fine Chemicals) (2.4 ml) was added, and the reaction mixture was subjected to vortex mixing for 30 s followed by centrifugation at 3,200 × *g* for 10 min at 4°C to separate the different phases. A 2.4-ml volume of the aqueous layer (top layer) was added to 3.2 ml of hot phenol-chloroform (Sigma) (5:1, 75°C), subjected to vortex mixing for 3 min, and centrifuged at 3,200 × *g* for 10 min at 4°C. An equal volume of the aqueous phase was added to 0.7 ml isopropanol in 4 separate microcentrifuge tubes, inverted, and left at room temperature for 10 min. The tube was processed by microcentrifugation for 25 min at maximum speed at 4°C, the supernatant was removed, and the pellet was washed in 1 ml cold 75% ethanol at maximum speed at 4°C. The supernatant was removed, the pellet was air dried for 10 min, and the pellet was rehydrated in 20 μl of nuclease-free water by heating at 55°C for 5 min. The four parallel samples were combined to reach a final volume of 80 μl. TRIzol (Ambion) (1.2 ml) was added, and the reaction mixture was subjected to vortex mixing for 15 s and was then allowed to stand at room temperature for 5 min. Chloroform (0.24 ml) was added to the tube, and the tube was mixed by inversion for 15 s, allowed to stand for 2 min, and pelleted by microcentrifugation at maximum speed for 15 min at 4°C. The aqueous layer (0.7 ml) was mixed with an equal of isopropanol, inverted for mixing, and allowed to stand at room temperature for 10 min. The tubes were processed by microcentrifugation for 25 min at maximum speed and 4°C, the supernatant was removed, and the pellet was washed in 1 ml 75% ethanol at 4°C. The pellet was recollected by centrifugation, the supernatant was discarded, and the pellet was air dried for 10 min at room temperature. Finally, the pellet was resuspended in 80 μl of nuclease-free water by heating at 55°C for 5 min. Isolated RNA was treated with DNase (Turbo DNA-free kit; Ambion). RNA was reextracted from the DNase reaction mixture following the protocol described above starting at the TRIzol step.

### Quantitative reverse transcriptase PCR.

To generate a standard curve, the *hag* transcript was transcribed *in vitro* using a PCR-derived template with a T7 promoter and primer pair 5373/5374. The template was used with a MEGAscript T7 kit (Invitrogen) to generate *hag* transcripts following the protocol described by the manufacturer. The *in vitro*-transcribed *hag* transcript was spiked into the *Δhag* lysate (DS1677) at a concentration of 500 ng/μl in a total volume of 80 μl prior to the addition of chloroform at the beginning of the RNA purification protocol and was reextracted as described above. The amount recovered was presumed to be the same as the input, less a proportional loss due to recovery from lysates, similarly to the loss expected for native samples.

For both standard curve and native samples, cDNA was generated following the manufacturer protocol for a High Capacity cDNA reverse transcriptase kit (Invitrogen). Quantitative PCR was performed with primer pair 1564/1565 (71) and either cDNA or a negative control (the samples that were processed in parallel but were not treated with reverse transcriptase) using SYBR Select Master Mix (Applied Biosystems) with an Applied Bioscience StepOnePlus qRT-PCR machine (Life Technologies). The *hag* standard curve was serially diluted 9 times prior to being added to the quantitative PCR. The number of *hag* transcripts was determined using a standard curve (as described above) and the following calculations.

The number of *hag* transcript molecules per comparative threshold cycle (*C_T_*) value for the standard was determined using a calculator for quantifying the number of copies of a template (http://cels.uri.edu/gsc/cndna.html).

### Calculating *hag* transcripts per cell.

The comparative *C_T_* value determined for each sample was compared to the corresponding standard curve to determine the number of transcripts in that sample per nanogram of RNA. The number of transcripts per cell was calculated by the following equations: (transcript amount/nanogram) * (total nanograms of RNA isolated) = total transcripts; total transcripts/CFU = number of transcripts/cell.

## References

[1] Sturani E, Datta P, Hughes M, Gest H 1963 Regulation of enzyme activity by specific reversal of feedback inhibition. Science 141:1053. doi:10.1126/science.141.3585.1053. 14043347

[2] Woolfolk CA, Stadtman ER 1964 Cumulative feedback inhibition in the multiple end product regulation of glutamine synthetase activity in *Escherichia coli*. Biochem Biophys Res Commun 17:313–319. doi:10.1016/0006-291X(64)90003-8.

[3] Nester EW, Ra J 1966 Control of aromatic acid biosynthesis in Bacillus subtilis: sequential feedback inhibition. J Bacteriol 91:1594–1598. 4956345 10.1128/jb.91.4.1594-1598.1966 PMC316083

[4] Yates JL, Arfsten AE, Nomura M 1980 *In vitro* expression of *Escherichia coli* ribosomal protein genes: autogenous inhibition of translation. Proc Natl Acad Sci U S A 77:1837–1841. doi:10.1073/pnas.77.4.1837. 6445562 PMC348603

[5] Johnsen M, Christensen T, Dennis PP, Fiil NP 1982 Autogenous control: ribosomal protein L10-12 complex binds to the leader sequence of its mRNA. EMBO J 1:999–004. doi:10.1002/j.1460-2075.1982.tb01284.x. 6765237 PMC553148

[6] Kilmury SLN, Burrows LL 2016 Type IV pilins regulate their own expression via direct intramembrane interactions with the sensor kinase PilS. Proc Natl Acad Sci U S A 113:6017–6022. doi:10.1073/pnas.1512947113. 27162347 PMC4889343

[7] Muhkerjee S, Yakhnin H, Kysela D, Sokoloski J, Babitzke P, Kearns DB 2011 CsrA-FliW interaction governs flagellin homeostasis and a checkpoint on flagellar morphogenesis in *Bacillus subtilis*. Mol Microbiol 82:447–461. doi:10.1111/j.1365-2958.2011.07822.x. 21895793 PMC3192257

[8] Leifson E 1951 Staining, shape, and arrangement of bacterial flagella. J Bacteriol 62:377–389. 14897809 10.1128/jb.62.4.377-389.1951 PMC386144

[9] Berg HC, Anderson RA 1973 Bacteria swim by rotating their flagellar filaments. Nature 245:380–382. doi:10.1038/245380a0. 4593496

[10] Silverman M, Simon M 1974 Flagellar rotation and the mechanism of bacterial motility. Nature 249:73–74. doi:10.1038/249073a0. 4598030

[11] Asakura S, Eguchi G, Iino T 1968 Unidirectional growth of *Salmonella* flagella *in vitro*. J Mol Biol 35:227–236. doi:10.1016/S0022-2836(68)80050-6. 5760562

[12] LaVallie ER, Stahl ML 1989 Cloning of the flagellin gene from *Bacillus subtilis* and complementation studies of an *in vitro*-derived deletion mutation. J Bacteriol 171:3085–3094. doi:10.1128/jb.171.6.3085-3094.1989. 2498283 PMC210019

[13] Minamino T, Macnab RM 1999 Components of the *Salmonella* flagellar export apparatus and classification of export substrates. J Bacteriol 181:1388–1394. 10049367 10.1128/jb.181.5.1388-1394.1999 PMC93525

[14] Hirano T, Minamino T, Namba K, Macnab RM 2003 Substrate specificity classes and the recognition signal for Salmonella type III flagellar export. J Bacteriol 185:2485–2492. doi:10.1128/JB.185.8.2485-2492.2003. 12670972 PMC152621

[15] Emerson SU, Tokuyasu K, Simon MI 1970 Bacterial flagella: polarity of elongation. Science 169:190–192. doi:10.1126/science.169.3941.190. 4987789

[16] Ikeda T, Oosawa K, Hotani H 1996 Self-assembly of the filament capping protein, FliD, of bacterial flagella into an annular structure. J Mol Biol 259:679–686. doi:10.1006/jmbi.1996.0349. 8683574

[17] Auvray F, Thomas J, Fraser GM, Hughes C 2001 Flagellin polymerisation control by cytosolic export chaperone. J Mol Biol 308:221–229. doi:10.1006/jmbi.2001.4597. 11327763 PMC2528291

[18] Renault TT, Abraham AO, Bergmiller T, Paradis G, Rainville S, Charpentier E, Guet CC, Tu Y, Namba K, Keener JP, Minamino T, Erhardt M 2017 Bacterial flagella grow through an injection-diffusion mechanism. Elife 6:e23136. doi:10.7554/eLife.23136. 28262091 PMC5386592

[19] Macnab RM 2003 How bacteria assemble flagella. Annu Rev Microbiol 57:77–100. doi:10.1146/annurev.micro.57.030502.090832. 12730325

[20] Smith DR, Chapman MR 2010 Economical evolution: microbes reduce the synthetic cost of extracellular proteins. mBio 1:e00131-10. doi:10.1128/mBio.00131-10. 20824102 PMC2932507

[21] Chevance FFV, Hughes KT 2008 Coordinating assembly of a bacterial macromolecular machine. Nat Rev Microbiol 6:455–465. doi:10.1038/nrmicro1887. 18483484 PMC5963726

[22] Titz B, Rajagopala SV, Ester C, Häuser R, Uetz P 2006 Novel conserved assembly factor of the bacterial flagellum. J Bacteriol 188:7700–7706. doi:10.1128/JB.00820-06. 16936039 PMC1636259

[23] Mukherjee S, Babitzke P, Kearns DB 2013 FliW and FliS function independently to control cytoplasmic flagellin levels in *Bacillus subtilis*. J Bacteriol 195:297–306. doi:10.1128/JB.01654-12. 23144244 PMC3553831

[24] Altegoer F, Mukherjee S, Steinchen W, Bedrunka P, Linne U, Kearns DB, Bange G 2018 FliS/flagellin/FliW heterotrimer couples type III secretion and flagellin homeostasis. Sci Rep 8:11552. doi:10.1038/s41598-018-29884-8. 30068950 PMC6070490

[25] Yakhnin H, Pandit P, Petty TJ, Baker CS, Romeo T, Babitzke P 2007 CsrA of *Bacillus subtilis* regulates translation initiation of the gene encoding the flagellin protein (*hag*) by blocking the ribosome binding. Mol Microbiol 64:1605–1620. doi:10.1111/j.1365-2958.2007.05765.x. 17555441

[26] Mukherjee S, Oshiro RT, Yakhnin H, Babitzke P, Kearns DB 2016 FliW antagonizes CsrA RNA binding by a noncompetitive allosteric mechanism. Proc Natl Acad Sci U S A 113:9870–9875. doi:10.1073/pnas.1602455113. 27516547 PMC5024615

[27] Altegoer F, Rensing S, Bange G 2016 Structural basis for the CsrA-dependent modulation of translation initiation by an ancient regulatory protein. Proc Natl Acad Sci U S A 113:10168–10173. doi:10.1073/pnas.1602425113. 27551070 PMC5018767

[28] Pigolotti S, Krishna S, Jensen MH 2007 Oscillation patterns in negative feedback loops. Proc Natl Acad Sci U S A 104:6533–6537. doi:10.1073/pnas.0610759104. 17412833 PMC1871820

[29] Sze CW, Morado DR, Liu J, Charon NW, Xu H, Li C 2011 Carbon storage regulator A (CsrA_Bb_) is a repressor of *Borrelia burgdorferi* flagellin protein FlaB. Mol Microbiol 82:851–864. doi:10.1111/j.1365-2958.2011.07853.x. 21999436 PMC3212630

[30] Dugar G 2016 The CsrA-FliW network controls polar localization of the dual-function flagellin mRNA in *Campylobacter jejuni*. Nat Commun 7:11667. doi:10.1038/ncomms11667. 27229370 PMC4894983

[31] Radomska KA, Ordoñez SR, Wösten MMSM, Wagenaar JA, van Putten JPM 2016 Feedback control of *Campylobacter jejuni* flagellin levels through reciprocal binding of FliW to flagellin and the global regulator CsrA. Mol Microbiol 102:207–220. doi:10.1111/mmi.13455. 27353476

[32] Yamaguchi Y, Park J, Inouye M 2011 Toxin-antitoxin systems in bacteria and archaea. Annu Rev Genet 45:61–79. doi:10.1146/annurev-genet-110410-132412. 22060041

[33] Blair KM, Turner L, Winkelman JT, Berg HC, Kearns DB 2008 A molecular clutch disables flagella in the *Bacillus subtilis* biofilm. Science 320:1636–1638. doi:10.1126/science.1157877. 18566286

[34] Wang F 2017 A structural model of flagellar filament switching across multiple bacterial species. Nat Commun 8:960. doi:10.1038/s41467-017-01075-5. 29038601 PMC5643327

[35] Guttenplan SB, Shaw S, Kearns DB 2013 The cell biology of peritrichous flagella in *Bacillus subtilis*. Mol Microbiol 87:211–229. doi:10.1111/mmi.12103. 23190039 PMC3538361

[36] Mukherjee S 2015 Adaptor-mediated Lon proteolysis restricts *Bacillus subtilis* hyperflagellation. Proc Natl Acad Sci U S A 11:250–255. doi:10.1073/pnas.1417419112. PMC4291670 25538299

[37] Samatey FA, Matsunami H, Imada K, Nagashima S, Shaikh TR, Thomas DR, Chen JZ, DeRosier DJ, Kitao A, Namba K 2004 Structure of the bacterial flagellar hook and implication for the molecular universal joint mechanism. Nature 431:1062–1068. doi:10.1038/nature02997. 15510139

[38] Courtney CR, Cozy LM, Kearns DB 2012 Molecular characterization of the flagellar hook in *Bacillus subtilis*. J Bacteriol 194:4619–4629. doi:10.1128/JB.00444-12. 22730131 PMC3415477

[39] Homma M, Fujita H, Yamaguchi S, Iino T 1984 Excretion of unassembled flagellin by *Salmonella typhimurium* mutants deficient in hook-associated proteins. J Bacteriol 159:1056–1059. 6384179 10.1128/jb.159.3.1056-1059.1984 PMC215768

[40] Hughes KT, Gillen KL, Semon MJ, Karlinsey JE 1993 Sensing structural intermediates in bacterial flagellar assembly by export of a negative regulator. Science 262:1277–1280. doi:10.1126/science.8235660. 8235660

[41] Kutsukake K 1994 Excretion of the anti-sigma factor through a flagellar substructure couples flagellar gene expression with flagellar assembly in Salmonella typhimurium. Mol Gen Genet 243:605–612. 8028576 10.1007/BF00279569

[42] Barillà D, Caramori T, Galizzi A 1994 Coupling of flagellin gene transcription to flagellar assembly in *Bacillus subtilis*. J Bacteriol 176:4558–4564. doi:10.1128/jb.176.15.4558-4564.1994. 8045886 PMC196275

[43] Calvo RA, Kearns DB 2015 FlgM is secreted by the flagellar export apparatus in *Bacillus subtilis*. J Bacteriol 197:81–91. doi:10.1128/JB.02324-14. 25313396 PMC4288692

[44] Gutierrez P, Li Y, Osborne MJ, Pomerantseva E, Liu Q, Gehring K 2005 Solution structure of the carbon storage regulator protein CsrA from *Escherichia coli*. J Bacteriol 187:3496–3501. doi:10.1128/JB.187.10.3496-3501.2005. 15866937 PMC1112004

[45] Mercante J, Edwards AN, Dubey AK, Babitzke P, Romeo T 2009 Molecular geometry of CsrA (RsmA) binding to RNA and its implications for regulated expression. J Mol Biol 392:511–528. doi:10.1016/j.jmb.2009.07.034. 19619561 PMC2735826

[46] Adler HI, Fisher WD, Cohen A, Hardigree AA 1967 Miniature *Escherichia coli* cells deficient in DNA. Proc Natl Acad Sci U S A 57:321–326. doi:10.1073/pnas.57.2.321. 16591472 PMC335508

[47] de Boer PAJ, Crossley RE, Rothfield LI 1989 A division inhibitor and a topological specificity factor coded for the minicell locus determine proper placement of the division septum in *E. coli*. Cell 56:641–649. doi:10.1016/0092-8674(89)90586-2. 2645057

[48] Bi E, Lutkenhaus J 1993 Cell division inhibitors SulA and MinCD prevent formation of the FtsZ ring. J Bacteriol 175:1118–1125. doi:10.1128/jb.175.4.1118-1125.1993. 8432706 PMC193028

[49] Jones CJ, Macnab RM, Okino H, Aizawa SI 1990 Stoichiometric analysis of the flagellar hook-(basal-body) complex of *Salmonella typhimurium*. J Mol Biol 212:377–387. doi:10.1016/0022-2836(90)90132-6. 2181149

[50] Wei BL, Brun-Zinkernagel AM, Simecka JW, Prüss BM, Babitzke P, Romeo T 2001 Positive regulation of motility and *flhDC* expression by the RNA-binding protein CsrA of Escherichia coli. Mol Microbiol 40:245–256. doi:10.1046/j.1365-2958.2001.02380.x. 11298291

[51] Figueroa-Bossi N, Schwartz A, Guillemardet B, D'Heygere F, Bossi L, Boudvillain M 2014 RNA remodeling by bacterial global regulator CsrA promotes Rho-dependent transcription termination. Genes Dev 28:1239–1251. doi:10.1101/gad.240192.114. 24888591 PMC4052769

[52] Esquerré T, Bouvier M, Turlan C, Carpousis AJ, Girbal L, Cocaign-Bousquet M 2016 The Csr system regulates genome-wide mRNA stability and transcription and thus gene expression in Escherichia coli. Sci Rep 6:25057. doi:10.1038/srep25057. 27112822 PMC4844966

[53] Potts AH, Leng Y, Babitzke P, Romeo T 2018 Examination of Csr regulatory circuitry using epistasis analysis with RNA-seq (Epi-seq) confirms that CsrD affects gene expression via CsrA, CsrB and CsrC. Sci Rep 8:5373. doi:10.1038/s41598-018-23713-8. 29599472 PMC5876332

[54] Potts AH, Vakulskas CA, Pannuri A, Yakhnin H, Babitzke P, Romeo T 2017 Global role of the bacterial post-transcriptional regulator CsrA revealed by integrated transcriptomics. Nat Commun 8:1596. doi:10.1038/s41467-017-01613-1. 29150605 PMC5694010

[55] Aizenman E, Engelberg-Kulka H, Glaser G 1996 An *Escherichia coli* chromosomal “addiction module” regulated by guanosine 3’, 5’-bispyrophosphate: a model for programmed bacterial cell death. Proc Natl Acad Sci U S A 93:6059–6063. doi:10.1073/pnas.93.12.6059. 8650219 PMC39188

[56] Gotfredsen M, Gerdes K 1998 The *Escherichia coli relBE* genes belong to a new toxin-antitoxin gene family. Mol Microbiol 29:1065–1076. doi:10.1046/j.1365-2958.1998.00993.x. 9767574

[57] Han J-S, Lee JJ, Anandan T, Zeng M, Sripathi S, Jahng WJ, Lee SH, Suh J-W, Kang C-M 2010 Characterization of a chromosomal toxin-antitoxin, Rv1102c-Rv1103c system in *Mycobacterium tuberculosis*. Biochem Biophys Res Commun 400:293–298. doi:10.1016/j.bbrc.2010.08.023. 20705052

[58] Marianovsky I, Aizenman E, Engelberg-Kulka H, Glaser G 2001 The regulation of the *Escherichia coli mazEF* promoter involves an unusual alternating palindrome. J Biol Chem 276:5975–5984. doi:10.1074/jbc.M008832200. 11071896

[59] Pedersen K, Zavialov AV, Pavlov MY, Elf J, Gerdes K, Ehrenberg M 2003 The bacterial toxin RelE displays codon-specific cleaved of mRNAs in the ribosomal A site. Cell 112:131–140. doi:10.1016/S0092-8674(02)01248-5. 12526800

[60] Overgaard M, Borch J, Jørgensen MG, Gerdes K 2008 Messenger RNA interferase RelE controls *relBE* transcription by conditional cooperativity. Mol Microbiol 69:841–857. doi:10.1111/j.1365-2958.2008.06313.x. 18532983

[61] Collins TJ 2007 ImageJ for microscopy. Biotechniques 43:25–30. doi:10.2144/000112517. 17936939

[62] Konkol MA, Blair KM, Kearns DB 2013 Plasmid-encoded ComI inhibits competence in the ancestral 3610 strain of *Bacillus subtilis*. J Bacteriol 195:4085–4093. doi:10.1128/JB.00696-13. 23836866 PMC3754741

[63] Yasbin RE, Young FE 1974 Transduction in *Bacillus subtilis* by bacteriophage SPP1. J Virol 14:1343–1348. 4214946 10.1128/jvi.14.6.1343-1348.1974 PMC355660

[64] Gibson DG, Young L, Chuang R-Y, Venter JC, Hutchison CA, Smith HO 2009 Enzymatic assembly of DNA molecules up to several hundred kilobases. Nat Methods 6:343–345. doi:10.1038/nmeth.1318. 19363495

[65] Bisson-Filho AW, Hsu Y-P, Squyres GR, Kuru E, Wu F, Jukes C, Sun Y, Dekker C, Holden S, VanNieuwenhze MS, Brun YV, Garner EC 2017 Treadmilling of FtsZ filaments drives peptidoglycan synthesis and bacterial cell division. Science 355:739–743. doi:10.1126/science.aak9973. 28209898 PMC5485650

[66] Bendezu FO, Hale CA, Bernhardt TG, de Boer PA 2009 RodZ (YfgA) is required for proper assembly of the MreB actin cytoskeleton and cell shape in *E. coli*. EMBO J 28:193–204. doi:10.1038/emboj.2008.264. 19078962 PMC2637328

[67] Rajkovic A, Hummels KR, Witzky A, Erickson S, Gafken PR, Whitelegge JP, Faull KF, Kearns DB, Ibba M 2016 Translation control of swarming proficiency in *Bacillus subtilis* by 5-amino-pentanolylated elongation factor P. J Biol Chem 291:10976–10985. doi:10.1074/jbc.M115.712091. 27002156 PMC4900249

[68] Rajendren S, Manning AC, Al-Awadi H, Yamada K, Takagi Y, Hundley HA 2018 A protein-protein interaction underlies the molecular basis for substrate recognition by an adenosine-to-inosine RNA-editing enzyme. Nucleic Acids Res 46:9647–9659. doi:10.1093/nar/gky800. 30202880 PMC6182170

[69] Yakhnin AV, Yakhnin H, Babitzke P 2012 Gel mobility shift assays to detect protein-RNA interactions. Methods Mol Biol 905:201–211. doi:10.1007/978-1-61779-949-5_12. 22736005 PMC4687016

[70] Cozy LM, Phillips AM, Calvo RA, Bate AR, Hsueh Y-H, Bonneau R, Eichenberger P, Kearns DB 2012 SlrA/SinR/SlrR inhibits motility gene expression upstream of the hypersensitive and hysteretic switch at the level of σ^D^ in *Bacillus subtilis*. Mol Microbiol 83:1210–1228. doi:10.1111/j.1365-2958.2012.08003.x. 22329926 PMC3303961

[71] Phillips AM, Calvo RA, Kearns DB 2015 Functional activation of the flagellar type III secretion export apparatus. PLoS Genet 11:e1005443. doi:10.1371/journal.pgen.1005443. 26244495 PMC4526659

[72] Doan T, Marquis KA, Rudner DZ 2005 Subcellular localization of a sporulation membrane protein is achieved through a network of interactions along and across the septum. Mol Microbiol 55:1767–1781. doi:10.1111/j.1365-2958.2005.04501.x. 15752199

[73] Patrick JE, Kearns DB 2008 MinJ (YvjD) is a topological determinant of cell division in *Bacillus subtilis*. Mol Microbiol 70:1166–1179. doi:10.1111/j.1365-2958.2008.06469.x. 18976281

