# Supplementary material for: Robust Stoichiometry of FliW-CsrA Governs Flagellin Homeostasis and Cytoplasmic Organization in Bacillus subtilis
Source: mBio. 2019 May 21;10(3):e00533-19. doi: 10.1128/mBio.00533-19 (PMC6529632; doi:10.1128/mBio.00533-19)
Supplement: FIG S1 [file mBio.00533-19-sf001.pdf]

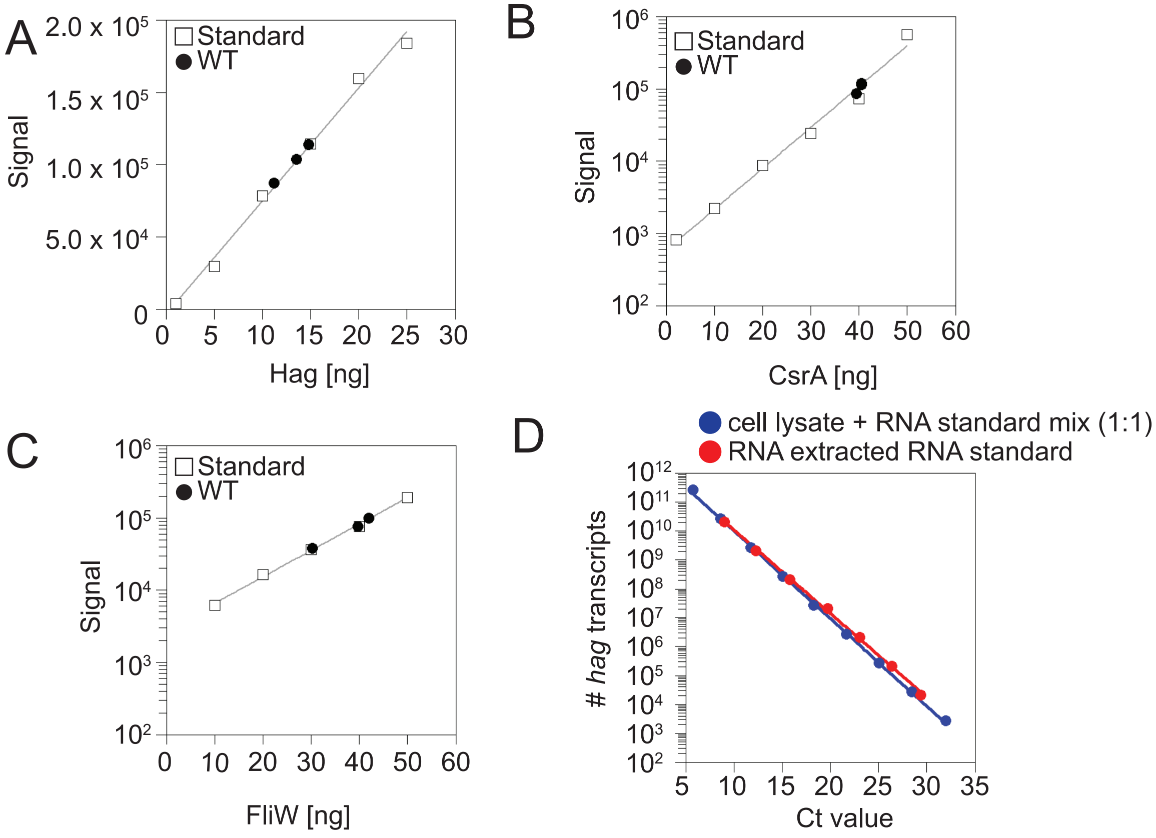

Figure S1. Standard curves used for quantitative Western blot and qRT-PCR. Plotted standard curves for the quantitative Western blot analysis of Hag (A), CsrA (B), and FliW (C) with nanograms (ng) on the x-axis and signal on the y-axis. Each dot represents a replicate for wild type and signal intensity within the standard curve. Panel D) Plotted standard curve used for qRT-PCR with Ct value on the x-acis and the number of hag transcripts on the y-axis. Blue dots represent the standard curve generated when the in vitro transcribed hag transcript was mixed at a 1:1: ratio with RNA extracted from the hag deletion background (DS1677). Red dots represent the standard curve generated when the in vitro transcribed hag transcript was added to the lysed hag deletion background and re-extracted (see methods for more detail).
