# Supplementary material for: Robust Stoichiometry of FliW-CsrA Governs Flagellin Homeostasis and Cytoplasmic Organization in Bacillus subtilis
Source: mBio. 2019 May 21;10(3):e00533-19. doi: 10.1128/mBio.00533-19 (PMC6529632; doi:10.1128/mBio.00533-19)
Supplement: TABLE S1 [file mBio.00533-19-st001.docx]

**Supplemental Table S1: Quantification of Hag, FliW, CsrA, and the *hag* transcript per cell**

| **Strain** | **Hag monomers ± Std. Dev.** | **FliW monomers ± Std. Dev.** | **CsrA dimers ± Std. Dev.** | ***hag transcript* ± Std. Dev** |
| --- | --- | --- | --- | --- |
| WT | 127,944 ± 6,460 | 9,111 ± 1,607 | 8,268 ± 1,565 | 22,114 ± 4,258 |
| *fliD* | 6,336 ± 830 | 5,328 ± 1,823 | 11,059 ± 3,078 | 138,383 ± 37,478 |
| *flgE* | 2,046 ± 272 | 5,750 ± 314 | 7,796 ± 1,107 | 465 ± 105 |
| *flgE flgM* | 7,659 ± 1,948 | 8,556 ± 1,643 | 8,354 ± 3,613 | 23,669 ± 5,170 |
| *flgE csrA* | 23,797 ± 5,372 | 12,237 ± 1,212 | N.D. | 14,207 ± 6,693 |
| *flgE flgM csrA* | 251,111 ± 144,764 | 14,381 ± 3,447 | N.D. | 86,509 ± 47,900 |
