# Supplementary material for: Robust Stoichiometry of FliW-CsrA Governs Flagellin Homeostasis and Cytoplasmic Organization in Bacillus subtilis
Source: mBio. 2019 May 21;10(3):e00533-19. doi: 10.1128/mBio.00533-19 (PMC6529632; doi:10.1128/mBio.00533-19)
Supplement: FIG S2 [file mBio.00533-19-sf002.pdf]

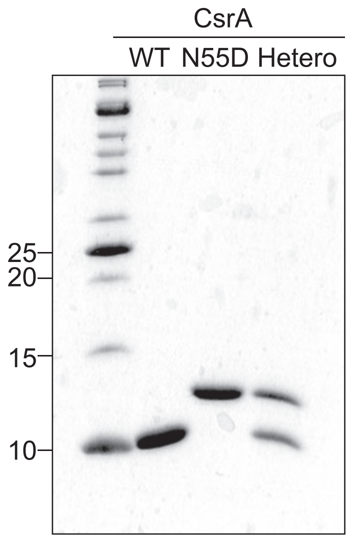

Figure S2. Purified CsrA constructs. Denaturing gel of purified CsrA constructs loaded in equimolar concentration stained with brilliant blue. The ratio of the two constructs, $CsrA^{WT}$-His_6 and $CsrA^{N55D}$-strep, that make up the $CsrA^{(Hetero)dimer}$ were determined by densitometry and were found to be at a 0.88:1 ratio.
