# Supplementary material for: Robust Stoichiometry of FliW-CsrA Governs Flagellin Homeostasis and Cytoplasmic Organization in Bacillus subtilis
Source: mBio. 2019 May 21;10(3):e00533-19. doi: 10.1128/mBio.00533-19 (PMC6529632; doi:10.1128/mBio.00533-19)
Supplement: FIG S3 [file mBio.00533-19-sf003.pdf]

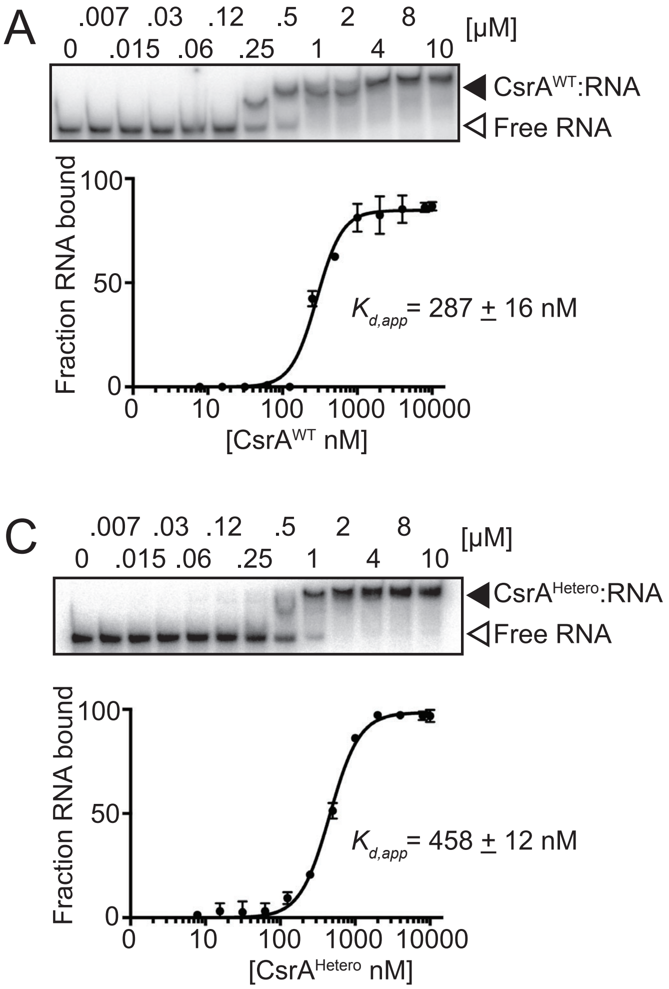

Figure S3. Binding affinity of CsrA Constructs and FliW to the hag transcript. Panel A-D, Top) RNA electrophoretic mobility shift assays were performed using the +1-100 region of the hag transcript and the indicated amounts of $CsrA^{WT}$-His_6,$CsrA^{N55D}$-strep, $CsrA^{Hetero(dimer)}$, and FliW. “Free” indicates the position of the unbound probe (open triangles). Panel A-D, Bottom) Calculated binding curves and $K_{d,apparent}$ ($K_{d,app}$) values for each construct listed above. $K_{d,app}$ values and standard deviation were calculated with at least three replicates.
