# Supplementary material for: Robust Stoichiometry of FliW-CsrA Governs Flagellin Homeostasis and Cytoplasmic Organization in Bacillus subtilis
Source: mBio. 2019 May 21;10(3):e00533-19. doi: 10.1128/mBio.00533-19 (PMC6529632; doi:10.1128/mBio.00533-19)
Supplement: FIG S4 [file mBio.00533-19-sf004.pdf]

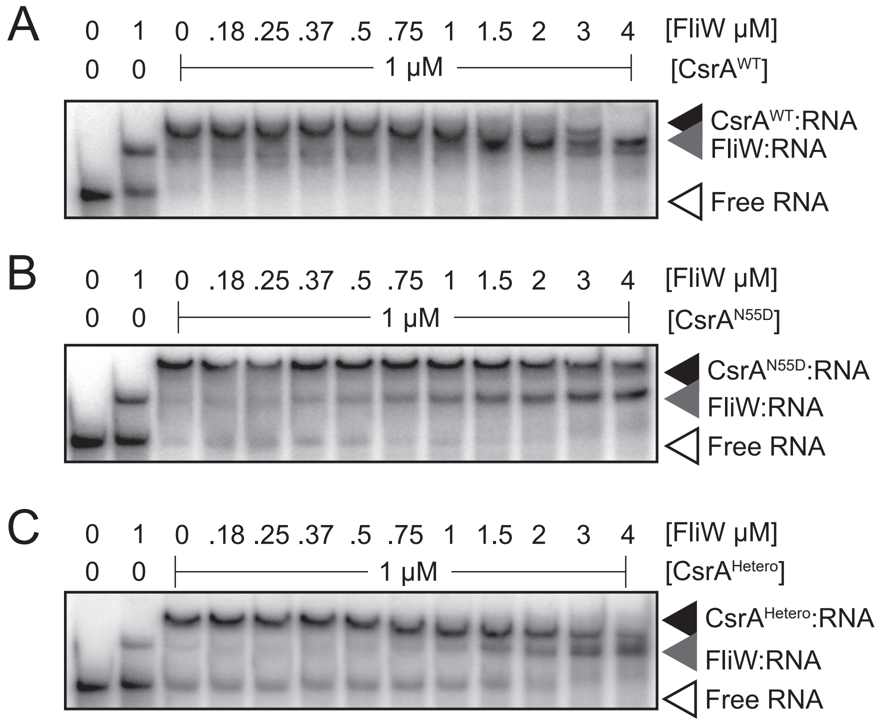

Figure S4. One molecule of FliW is necessary and sufficient to inhibit a dimer of CsrA. Panels A-C) RNA electrophoretic mobility shift assays (RNA EMSA) performed using the +1-100 region of the hag transcript and the indicated amounts of FliW in the presence of $CsrA^{WT}$-His_6 (panel A), $CsrA^{N55D}$-strep (panel B), or $CsrA^{Hetero(dimer)}$ (panel C). “Free” indicates the position of the unbound probe (open triangles). Black triangles indicate the position of the probe bound by CsrA. Gray triangles indicate the position of the probe bound by FliW. Each gel is a representative of experiments repeated in triplicate.
