# Supplementary material for: Robust Stoichiometry of FliW-CsrA Governs Flagellin Homeostasis and Cytoplasmic Organization in Bacillus subtilis
Source: mBio. 2019 May 21;10(3):e00533-19. doi: 10.1128/mBio.00533-19 (PMC6529632; doi:10.1128/mBio.00533-19)
Supplement: FIG S5 [file mBio.00533-19-sf005.pdf]

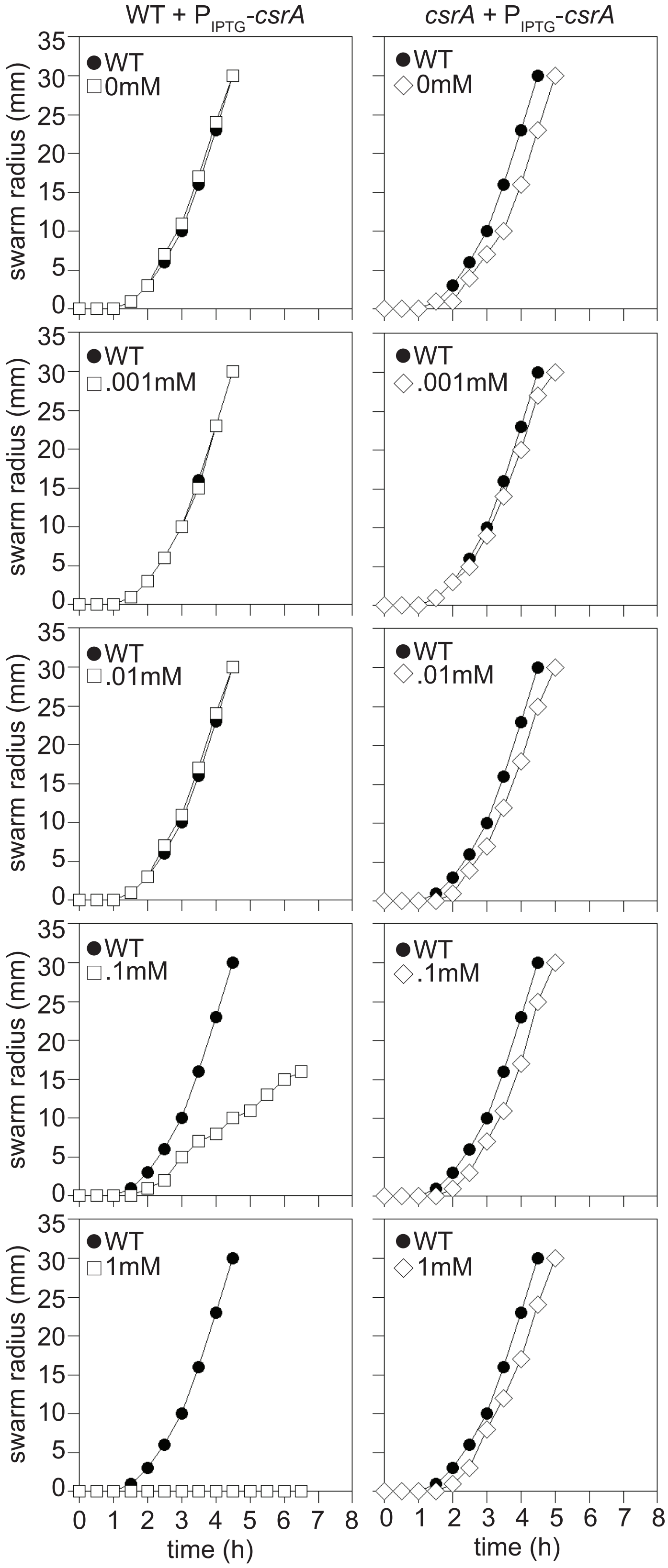

Figure S5. Induction of CsrA at an ectopic site inhibits swarming motility when the wild type copy of *csrA* is present. Quantitative swarm expansion assay of the strains $P_{hyspank}$-*csrA* (DS4940 - Left) and *csrA* $P_{hyspank}$-*csrA* (DK1522 - right) under varying concentrations of IPTG induction. Each point is the average of three replicates.
