# Supplementary material for: Robust Stoichiometry of FliW-CsrA Governs Flagellin Homeostasis and Cytoplasmic Organization in Bacillus subtilis
Source: mBio. 2019 May 21;10(3):e00533-19. doi: 10.1128/mBio.00533-19 (PMC6529632; doi:10.1128/mBio.00533-19)
Supplement: TABLE S2 [file mBio.00533-19-st002.docx]

**Supplemental Table S2. Quantification of the titration of CsrA dimers and FliW proteins per cell.**

| **strain** | **[IPTG mM]** | **CsrA dimers ± SD** | **FliW monomers ± SD** |
| --- | --- | --- | --- |
| Wild type | N.A. | 13,396 ± 1,740 | 12,592 ± 3,405 |
| *ΔcsrA amyE::Physpank-csrA* | 0 | 922 ± 695 | 13,312 ± 5,424 |
|  | 0.001 | 1,686 ± 1,348 | 15,702 ± 8,296 |
|  | 0.01 | 6,637 ± 1,376 | 20,788 ±1,532 |
|  | 0.1 | 14,259 ± 768 | 13,404 ± 2,511 |
|  | 1.0 | 12,511 ± 1,841 | 10,698 ± 859 |
| *amyE::Physpank-csrA* | 0 | 12,145 ± 1,108 | 19,143 ± 4,548 |
|  | 0.001 | 14,265 ± 1,746 | 20,796 ± 9,233 |
|  | 0.01 | 11,512 ± 3,170 | 20,916 ± 7,857 |
|  | 0.1 | 9,846 ± 1,784 | 12,114 ± 3,863 |
|  | 1.0 | 11,717 ± 3,251 | 11,455 ± 1,115 |
