# Supplementary material for: Robust Stoichiometry of FliW-CsrA Governs Flagellin Homeostasis and Cytoplasmic Organization in Bacillus subtilis
Source: mBio. 2019 May 21;10(3):e00533-19. doi: 10.1128/mBio.00533-19 (PMC6529632; doi:10.1128/mBio.00533-19)
Supplement: FIG S6 [file mBio.00533-19-sf006.pdf]

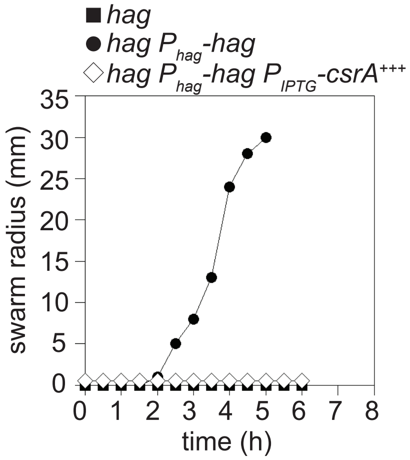

Figure S6. Inhibition of swarming motility by ectopic expression of CsrA is not due to a cis effect. Quantitative swarm expansion assay of the strains *hag* (DS1677 - squares), *hag* P_{hag}-*hag* (DS6235 - circles), and *hag* P_{hag}-*hag* $P_{hyspank}$-*csrA* (DK7072 – open diamonds). Each point is the average of three replicates. CsrA^{+++} indicates that CsrA was induced throughout growth and swarming by the addition of 1mM IPTG.
