# Supplementary material for: Robust Stoichiometry of FliW-CsrA Governs Flagellin Homeostasis and Cytoplasmic Organization in Bacillus subtilis
Source: mBio. 2019 May 21;10(3):e00533-19. doi: 10.1128/mBio.00533-19 (PMC6529632; doi:10.1128/mBio.00533-19)
Supplement: TABLE S3 [file mBio.00533-19-st003.docx]

**Supplemental Table S3. Quantification of the *hag* transcript under overexpression CsrA conditions**

| **Strain** | ***hag* Transcripts ± Std. Dev.** |
| --- | --- |
| Wild type | 23,500 ± 4,940 |
| *ΔcsrA amyE::Physpank-csrA* | 20,600 ± 4,930 |
| *amyE::Physpank-csrA* | 7,390 ± 2,090 |
| *sow3* | 22,700 ± 4,280 |
| *sow3 amyE::Physpank-csrA* | 25,900 ± 5,840 |
