# Supplementary material for: Robust Stoichiometry of FliW-CsrA Governs Flagellin Homeostasis and Cytoplasmic Organization in Bacillus subtilis
Source: mBio. 2019 May 21;10(3):e00533-19. doi: 10.1128/mBio.00533-19 (PMC6529632; doi:10.1128/mBio.00533-19)
Supplement: TABLE S4 [file mBio.00533-19-st004.docx]

**Supplemental Table S4: Primers and Plasmids**

| Primer | Sequence |
| --- | --- |
| 1483 | aggaggaattcatagtaaatcaggactccaaatgct |
| 1486 | ctcctggatccacagaactgacagatatgtgaatata |
| 1541 | aggaggctagcccgaaagaatcacccgaaaagtaa |
| 1544 | ctcctgcatgccggattgtctctgattttgttaatac |
| 1871 | aggaggaattcaaagcgggcagaatgtatttgaaatg |
| 1873 | aggaggtcgacaagcatccgattggaggagaatcat |
| 1907 | aggaggtcgacttgttacgtcaagatcattaactgaat |
| 4554 | ggaggagaatcatatgctagttttatcg |
| 4641 | tacgatttgaaataccatcgatttcat |
| 4972 | attcaggaagaagataaccgtgcagcagcgttatcc |
| 4973 | cgctgctgcacggttatcttcttcctgaatggtcaagtaaatttctttcctgtga |
| 5352 | tttttaagaattattaatttgatcttgatgaatcaactgctgaact |
| 5353 | atcaagatcaaattaataattcttaaaaaagataaacggacttacgacga |
| 5371 | cgacaaagcatccgattggtggagaatcatgctagttttatcgcg |
| 5372 | aaaactagcatgattctccaccaatcggatgctttgtcgt |
| 5373 | tcccgcgaaattaatacgactcactataggtaatgatgtagccgggaggag |
| 5374 | cacatatttttggaaaaaaatcttctgttttagat |
| 5434 | gtcgactctagaggatccccgcgggcagaatgtatttgaaatg |
| 5435 | tgaattcgagctcggtacccttgttacgtcaagatcattaactgaatgaaga |
| 5967 | aggagggtaccttgttagcagccggatc |
| 6285 | agggcacaagaacgtgcctta |
| 6286 | taaggcacgttcttgtgccct |
| 6315 | aggaggaattctcacttttcgaattgtggatgagaccagttggcacccttttcgaattgtggatgagaccagttggcacccttttcgaattgtggatgagaccagttggcacccttttcgaattgtggatgagaccaacctccaccaccgcctccaccaccctttttttgtgaggataatgcg |
| 6316 | aggagccatggctctagttttatcgcggaaaa |
| 6690 | tcccgcgaaattaatacgactcactataggtagccgggaggaggc |
| 6691 | aattctcattgttttgttcctccct |
|  |  |
| Plasmid | Genotype |
| pCSB9 | *P_T7_-CsrA-His_6_ amp* (25) |
| pDR244 | *Cre recombinase spec amp* (Gift from David Rudner) |
| pDR183 | *lacA::mls amp* (72) |
| pDP306 | *ΩΔflgE mls amp* (38) |
| pETDUET-1 | *P_T7_-His_6_ P_T7_-S-tag amp* (Novagen) |
| pJP87 | *ΩΔfliW mls amp* (7) |
| pKB142 | *lacA::P_hag_-hag^T209C^ mls* |
| pMiniMAD | *ori^BsTs^ amp mls* (73) |
| pNE4 | *amyE::Phag-hag^T209C^ spec amp* (33) |
| pRO25 | *ΩcsrA^N55D^ mls amp* |
| pRO30 | *ΩfliW^E71stop^ mls amp* |
| pRO33 | *ΩfliW^G139G^ mls amp* |
| pRO68 | *Ωhag^sow3^ mls amp* |
| pRO76 | *P_T7_-CsrA^N55D^-strep tag P_T7_-CsrA-His_6_ amp* |
| pRO77 | *P_T7_-CsrA^N55D^-strep tag amp* |
| pSG36 | *ΩΔfliWcsrA mls amp* (7) |
| pSM12 | *P_T7_-His_6_-SUMO-FliW amp* (7) |
| pSM56 | *P_T7_-His_6_-SUMO-Hag amp* (23) |
